# Iron Ore Tailings in Construction Materials: A Systematic Review of Composition and Water Absorption Performance

**DOI:** 10.3390/ma19204385

**Published:** 2026-10-19

**Authors:** Rivelino Neri Silva, Anderson Ravik dos Santos, Alberto José Corrêa de Souza, Keoma C. S. Defáveri, Patrícia Santiago de Oliveira Patrício, Wanna Carvalho Fontes

**Affiliations:** 1 Department of Civil Engineering, Morro do Cruzeiro University Campus, Federal University of Ouro Preto (UFOP), Ouro Preto 35400-000, Brazil; rivelino.silva@aluno.ufop.edu.br (R.N.S.); anderson.ravik@aluno.ufop.edu.br (A.R.d.S.); alberto.souza@aluno.ufop.edu.br (A.J.C.d.S.); keoma.silva@ufop.edu.br (K.C.S.D.); 2 IntechLab Laboratory, Chemistry Department, Federal University of Science and Innovation of Minas Gerais (UFCI-MG), Belo Horizonte 30510-000, Brazil; patricia@cefetmg.br; 3 Department of Urban Engineering, Morro do Cruzeiro University Campus, Federal University of Ouro Preto (UFOP), Ouro Preto 35400-000, Brazil

**Keywords:** iron ore tailings, sustainability, water absorption, building materials

## Abstract

**Highlights:**

**What are the main findings?**

- Water absorption varied widely (0.1–49.2%) across IOT-based construction materials.
- Full IOT substitution in cement blocks achieved low water absorption (4.6%).
- Polymer–IOT composites showed the lowest water absorption (0.1–0.4%).
- Ceramic performance varied broadly (0.4–49.2%), driven mainly by firing conditions.
- Matrix type, IOT content, and processing jointly determine water absorption.

**What are the implications of the main findings?**

- Optimized curing or sintering can mitigate water absorption in IOT products.
- IOT valorization supports circular construction and reduces industrial waste.
- Findings guide matrix selection for durable, low-absorption IOT-based materials.
- Standardized testing protocols are needed to enable cross-study comparisons.

**Abstract:**

Iron ore tailings (IOTs) are generated in massive volumes by mining, posing environmental risks through land occupation, dam failures, and heavy metal contamination, while construction faces growing pressure toward sustainable materials. This systematic review evaluates IOT valorization in construction materials, focusing on composition and water absorption performance. A structured search in Scopus^®^ identified experimental studies (2015–2025, English), excluding reviews, simulations, and non-IOT studies. Data were synthesized narratively by matrix type. The 24 studies reporting water absorption were also assessed for methodological quality using a bespoke six-criterion checklist, as no validated risk-of-bias tool exists for such experiments. Of 145 documents screened, 74 met the inclusion criteria. IOT content ranged from below 10 wt% to full substitution across cement-based, ceramic, geopolymer, alkali-activated, and polymer matrices. Water absorption varied accordingly: cement-based blocks with full substitution achieved 4.6%, bricks reached 18.0%, geopolymer/AAC composites ranged 13.0–21.0%, polymers achieved the lowest values (0.1–0.4%), and ceramics varied broadly (0.4–49.2%). Quality assessment classified 54.2% of studies as low risk and 45.8% as moderate risk, and inconsistent variability reporting was the main limitation. Performance depended on matrix type, IOT content, and processing, supporting circular IOT reuse in construction. This review received CAPES funding and was not registered.

## 1. Introduction

The rising demand for sustainable practices in the construction industry has driven increasing attention toward the valorization of industrial by-products [1,2,3]. Among these, iron ore tailings (IOTs), generated in vast quantities during iron ore processing, pose considerable environmental and social challenges if not properly managed. The large amounts of IOT are commonly disposed of in piles or tailings dams [4,5,6].

For instance, in Brazil, it is estimated that iron ore waste accounts for approximately 20–40% of the total mass of iron ore extracted [7]. In 2022, Brazil was responsible for 17.1% of global iron ore production [8]. Applying Matiolo et al.’s [7] estimates to this share suggests that Brazil generated approximately 84 to 168 million tons of iron ore waste in that year alone. In 2022, the global production of iron ore amounted to 2455.7 million tons, with Australia, Brazil, China, and India contributing 38.4%, 17.1%, 10.3%, and 10.1% of the total output, respectively [8].

On the other hand, proportionally large amounts of IOT are generated and are commonly disposed of in piles or tailing dams. Improper disposal of these tailings can result in severe environmental impacts, including heavy metal contamination of soil and water, acid mine drainage, and harm to aquatic ecosystems and biodiversity [9,10,11]. In this context, improved tailings management and the proper utilization of IOT are key factors in minimizing the associated environmental impacts. The construction industry has emerged as a promising alternative for the incorporation of IOT into building materials, due to its large scale and capacity to absorb substantial volumes of waste across a wide variety of applications. This strategy aligns with the growing global demand for sustainable construction practices and the development of environmentally responsible materials [12].

Given the environmental urgency surrounding the disposal of IOT and the growing interest in their reuse within the construction industry, a comprehensive literature review is a coherent way to consolidate existing knowledge, identify technological trends, and highlight research gaps. Despite the growing body of research on the reuse of IOT in civil construction, the available literature often focuses on general material types or selected case studies, lacking a consolidated analysis of how key variables (such as matrix type, IOT content, and incorporation function) relate to material performance. Previous reviews, such as Santos et al. [13], have examined the potential applications of IOT from specific disaster contexts, like the Mariana dam collapse, highlighting the types of materials and basic performance tests involved. Others, like Lara et al. [14], focused primarily on mapping the physical–chemical characteristics of IOT in relation to their applications, identifying challenges in heterogeneity and legislative barriers. A third review [15] emphasized compressive strength as the main property of interest and explored large-scale scenario analyses, particularly in the Brazilian context. Notably, these studies diverge considerably in scope and emphasis, ranging from disaster-driven case analyses to physicochemical characterization and compressive-strength-focused scenarios, reflecting the absence of a unified framework for evaluating the durability performance of IOT-based materials. However, none of these studies conducted a detailed and comparative evaluation of experimental studies over the last decade that specifically relates IOT content, incorporation role, matrix type, and their effects on key durability properties, particularly water absorption, which is critical for material longevity.

This review aims to fill this gap by offering a systematic and structured synthesis of experimental studies, supported by scientometric and qualitative data analysis. The approach enables identification of trends, knowledge gaps, and promising combinations for the development of IOT-based building materials, thereby contributing a more refined and application-oriented perspective to the existing body of knowledge and supporting the development of more sustainable construction practices. The findings indicate that water absorption performance is strongly governed by matrix type, IOT content, and processing conditions, with polymer- and cement-based systems consistently achieving the lowest absorption values, offering practical guidance for material selection in future applications of IOT-based construction materials.

## 2. Methodology

**Figure 1:**
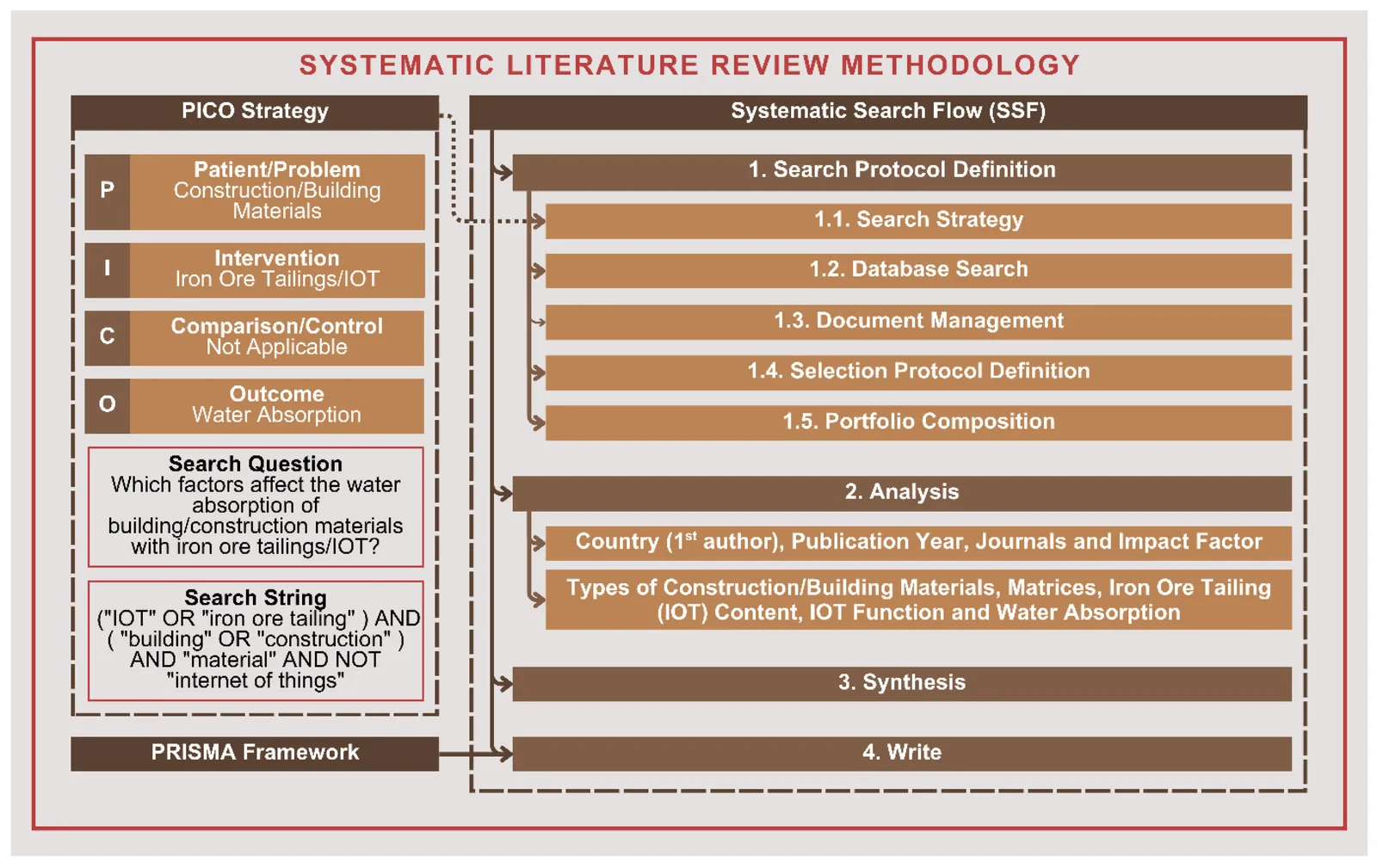
Systematic literature review methodology.

The systematic literature review was conducted according to the methodology summarized in Figure 1. The following data items were extracted from each included study: water absorption values (primary outcome) and, as additional variables, matrix type, IOT content, IOT functional role, country of the first author, publication year, journal, and journal impact factor (IF).

This study employed the Systematic Search Flow method [16], which establishes a structured process composed of research protocol, analysis, synthesis and writing. The PICO (Population, Intervention, Comparison, and Outcome) framework [17] was adapted to formulate the research question. In this context, the Population (P) refers to construction materials incorporating IOT, and the Intervention (I) refers to the incorporation process. The Comparison (C) component was not applicable, and the Outcome (O), water absorption, was intentionally omitted from the search string to avoid excluding studies that used different terminologies for similar effects. Finally, the PRISMA (Preferred Reporting Items for Systematic Reviews and Meta-Analyses) statement [18] was used to support transparent and complete reporting of the review process. This framework aligns particularly with Stage 4 of the Systematic Search Flow, ensuring that the decisions made during the search protocol definition and analysis phases are clearly documented. The File S1 provides structured guidance for reporting titles, abstracts, introductions, methodologies, results, and discussions in a systematic literature review.

**Figure 2:**
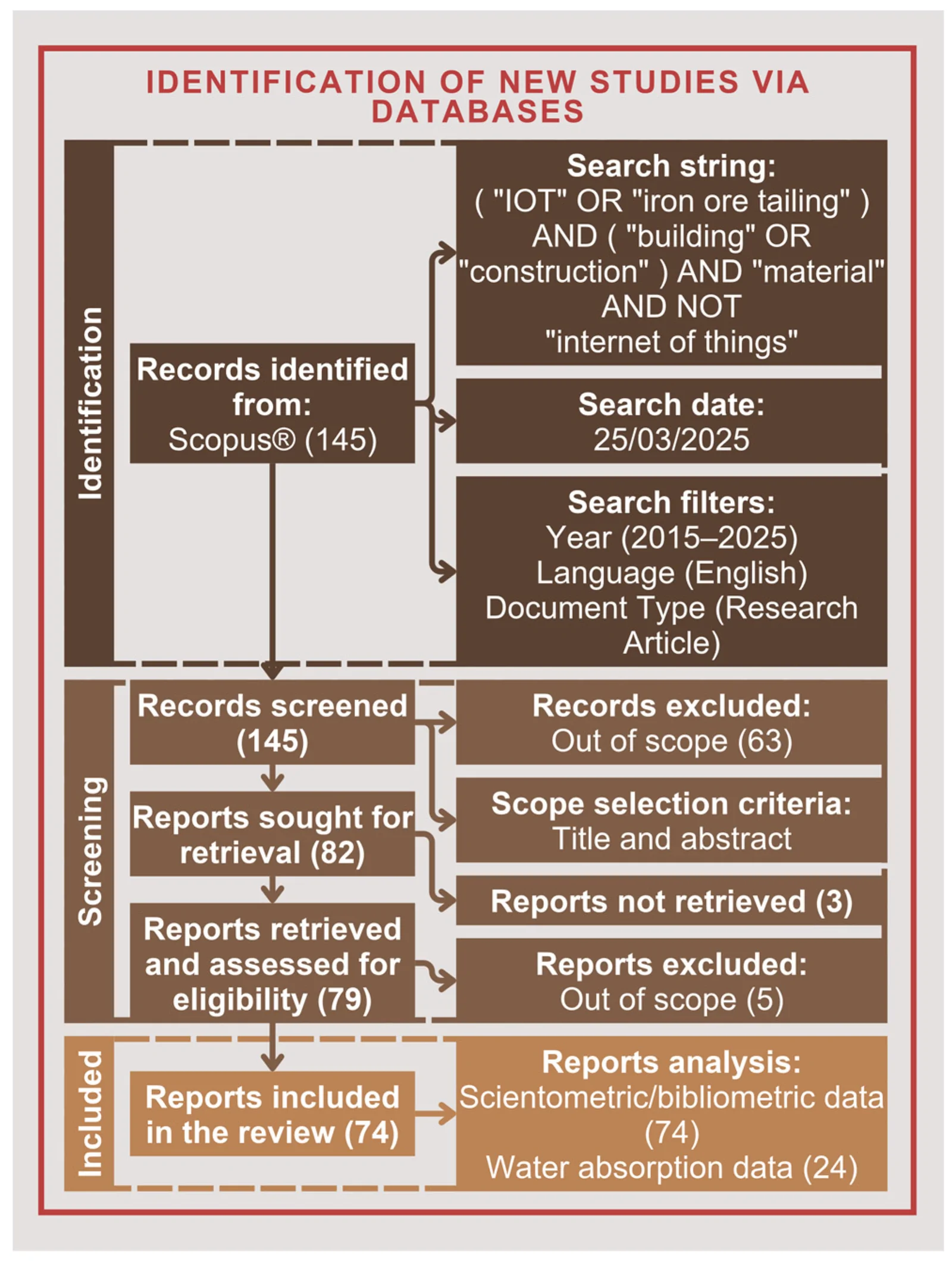
Bibliographic search conducted.

As also shown in Figure 2, a search was performed in the Scopus^®^ database on 25 March 2025, filtered by year (2015–2025), language (English), and document type (research articles). The query employed was (“IOT” OR “iron ore tailing”) AND (“building” OR “construction”) AND “material” AND NOT “internet of things” and returned 145 results. After screening the titles and abstracts, 63 studies were excluded for being out of scope, and three for lack of access. A full-text review of the remaining 79 studies led to the exclusion of an additional five studies. Thus, 74 articles were included in the final review.

Study selection (title/abstract and full-text screening) and data extraction were performed by a single reviewer, with no independent duplicate review, and extracted data were recorded in a standardized spreadsheet. Of those, 24 studies that reported experimental data on water absorption were selected for a focused analysis of that property. The inclusion criteria considered only experimental studies involving the application of IOT in construction materials. Review articles, computational studies, and studies focused solely on characterizing IOT without material application were excluded.

It should be noted that the water absorption values compiled from the selected studies were obtained using different testing standards (Table S2). Consequently, differences in methodological parameters may contribute to the variability observed among the reported values. Therefore, the graphical comparison presented herein should be interpreted as a synthesis of reported trends rather than as a direct comparison of measurements obtained under identical testing conditions.

No formal assessment of reporting bias (e.g., publication bias, selective outcome reporting) was conducted, as no statistical synthesis (meta-analysis) was performed. The potential for such bias among the included studies is acknowledged as a limitation of this review. This systematic review was not registered in a public registry (e.g., PROSPERO or OSF) prior to its conduct.

Extracted data were synthesized narratively, grouped primarily by the types of materials incorporating IOT and secondarily by matrix type, IOT content and functional role. No statistical pooling (meta-analysis) was performed given the heterogeneity of testing conditions and matrix systems across studies. Scientometric data were synthesized descriptively using frequency counts and cross-tabulations. When a study reported multiple content levels within the same predefined range, the data were counted only once in the frequency analysis.

Correlation between IOT content and water absorption (Tables S5–S7) and IOT chemical composition and water absorption (Tables S8 and S9) were assessed using both Pearson’s r and Spearman’s ρ. Given the scattered, non-normally distributed nature of water absorption data across studies, statistical significance was determined based on Spearman’s ρ, which is more robust to non-linear monotonic relationships and outliers than the Pearson coefficient. Categories with fewer than three data points, or with no variance in IOT content among available data points, were classified as “not applicable” for correlation analysis.

Zotero^®^ (v.7.0.15) was used for organizing references and automating citation management. Google Sheets^®^ supported the manual extraction and categorization of data from the selected articles, allowing classification based on variables such as matrix type, IOT content, and water absorption results. To visualize the relationships among categorical variables, OriginPro^®^ 2025b (v. 10.2.5.234) was used to generate Sankey diagrams. In cases where water absorption values were presented graphically rather than numerically, WebPlotDigitizer^®^ (v.5.0) enabled the extraction of approximate values from plots by calibrating axes and marking data points manually. A repeatability test was conducted on all data points extracted via this method (*n* = 49, across six studies), each extracted independently three times, where the median coefficient of variation was 0.36% and the mean absolute standard deviation was 0.024 percentage points, indicating high extraction repeatability (Supplementary Table S4). Finally, the water absorption scatter plot graphs were elaborated using OriginPro^®^ 2025b (v. 10.2.5.234).

Given the absence of a validated risk-of-bias tool for experimental studies in materials science and engineering (e.g., Cochrane RoB2 and ROBINS-I are designed for studies involving human participants), a bespoke six-criterion quality checklist was developed and applied to the 24 studies reporting water absorption data. One reviewer scored each study against six criteria: (i) IOT characterization reported (chemical and/or physical); (ii) use of a standardized, cited test method for water absorption; (iii) mix composition fully reported; (iv) number of replicates reported; (v) statistical variability reported (standard deviation, coefficient of variation, or error bars); and (vi) funding source or conflict-of-interest declared. Each criterion was scored as Yes (1), Partial (0.5), or No (0), yielding a total score from 0 to 6, classified as low risk (5–6), moderate risk (3–4.5), or high risk (0–2.5).

Quality assessment of the 24 studies reporting water absorption data indicated that 13 (54.2%) were classified as low risk and 11 (45.8%) as moderate risk, with no study classified as high risk (mean score 4.73/6). The most frequently unmet criterion was the reporting of statistical variability (standard deviation, coefficient of variation, or error bars), absent in 62.5% (15/24) of studies, followed by incomplete replicate reporting in 29.2% (7/24).

During the preparation of this work, the authors used OpenAI ChatGPT^®^ (GPT-4o), Google Gemini^®^ (v. 3.6), Microsoft Copilot^®^ (v. 155.0.4283.45), and Anthropic Claude^®^ (Sonnet 5) to enhance text clarity and coherence, and Anthropic Claude^®^ (Sonnet 5) supported risk of bias design. These tools were not used to generate data, figures, or study conclusions, nor to assist in data collection, analysis, or interpretation. After using these tools, the authors reviewed and edited the content as needed and take full responsibility for the content of the published article.

## 3. Results and Discussion

### 3.1. Scientific Production and Journal Impact Distribution

**Figure 3:**
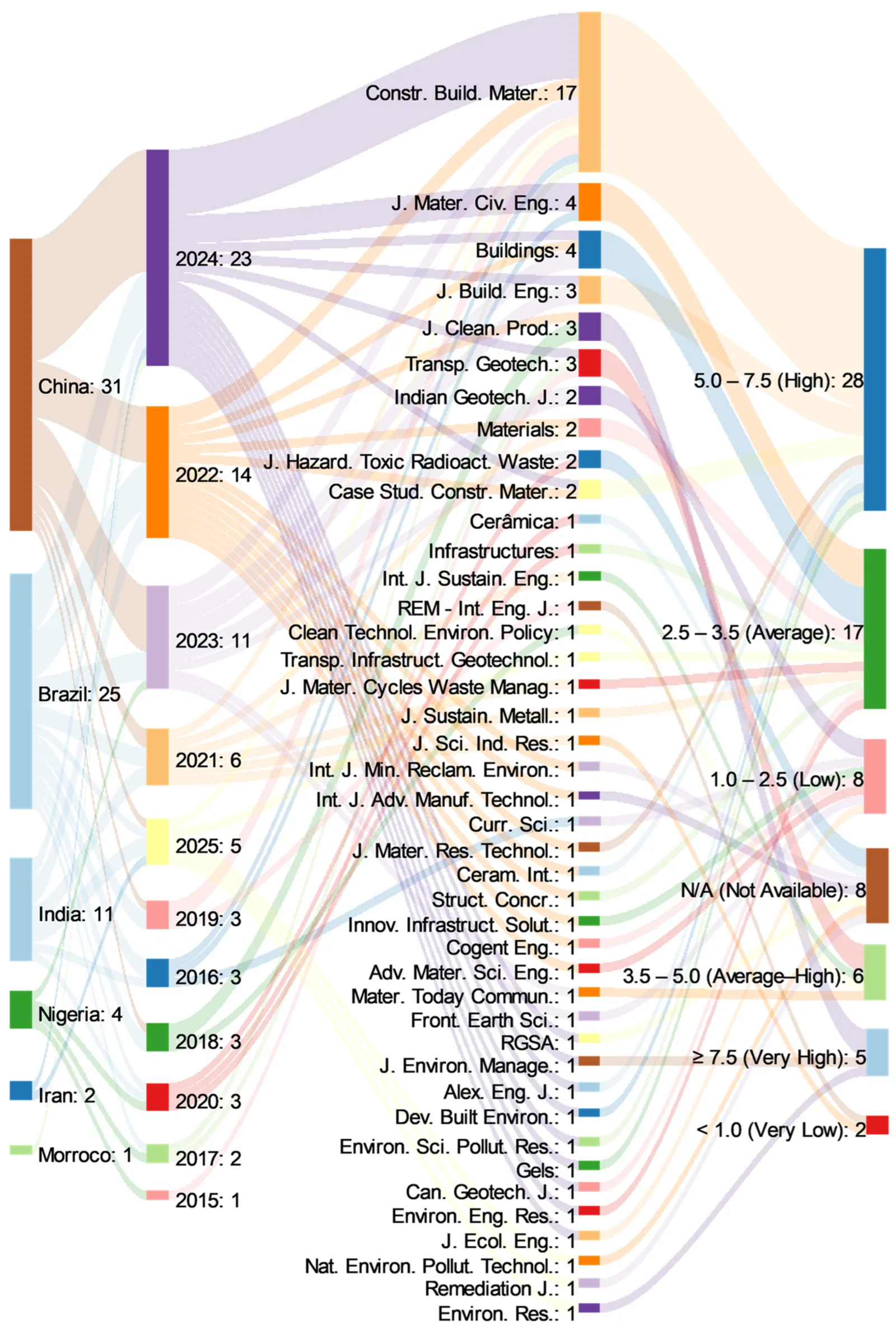
Flow of scientific publications from country to journal impact factors.

The scientific output in this field can be analyzed by examining the country of the first author, year of publication, journal, and the journal’s IF (based on the most recent available JCR data). These elements and their interrelations are depicted in Figure 3 and the data are displayed in Supplementary Table S1 (refer to electronic Supplementary Material).

Most of the publications originate from researchers based in China, Brazil, and India. This pattern can largely be attributed to the significant role these countries play in the global iron ore industry, consistently ranking among the top five producers worldwide. In 2024 alone, Brazil produced 440.0 million tons of iron ore, followed by China and India, which produced 270 million tons each [19].

Furthermore, in Brazil, two of the country’s most catastrophic industrial disasters, the Fundão Dam collapse in Mariana (2015) and the Brumadinho Dam collapse (2019), have heightened public awareness and pressure on industries to find safer and more sustainable solutions for IOT disposal [20,21]. These events catalyzed a surge in academic and industrial interest in repurposing IOT for construction applications, while also intensifying the demand for stricter enforcement of public policies, such as Brazil’s National Policy on Solid Waste [22]. Additionally, India and China’s large and growing populations generate increased demand for urban infrastructure. Within the context of the United Nations 2030 Agenda for Sustainable Development, both countries may be actively exploring eco-friendly alternatives in construction, such as the reuse of industrial by-products like IOT [23].

Although subject to minor fluctuations, the research trend on IOT-based building materials has shown steady growth over the past decade, reaching a peak in 2024 with 23 published papers, primarily from China and Brazil. This upward trajectory reflects a growing global interest in the development of sustainable construction materials incorporating IOT. The increase in research output may be driven by heightened awareness of the need for environmentally responsible waste management, combined with the demand for alternative building materials that align with the principles of sustainable development. Additionally, the topic has gained further relevance in light of the United Nations Sustainable Development Goals (SDGs), which have encouraged innovation in resource recovery and circular economy practices [23].

Many of the reviewed studies were published in Construction and Building Materials, a high-impact journal whose broad scope covering cement, polymers, recycled inputs, and alternative materials, aligns with research on IOT. This concentration reflects both thematic compatibility and the influence of publishing models. Journals like Construction and Building Materials, Journal of Cleaner Production, and Journal of Building Engineering offer open access and subscription options, which affect accessibility and cost, especially for researchers in countries with weaker currencies. Overall, most publications appear in journals focused on construction, materials science, or ceramics, with fewer addressing sustainability or geotechnology, underscoring the field’s emphasis on innovative applications of IOT.

### 3.2. From Waste to Product: Mapping IOT Integration in Construction Materials

**Figure 4:**
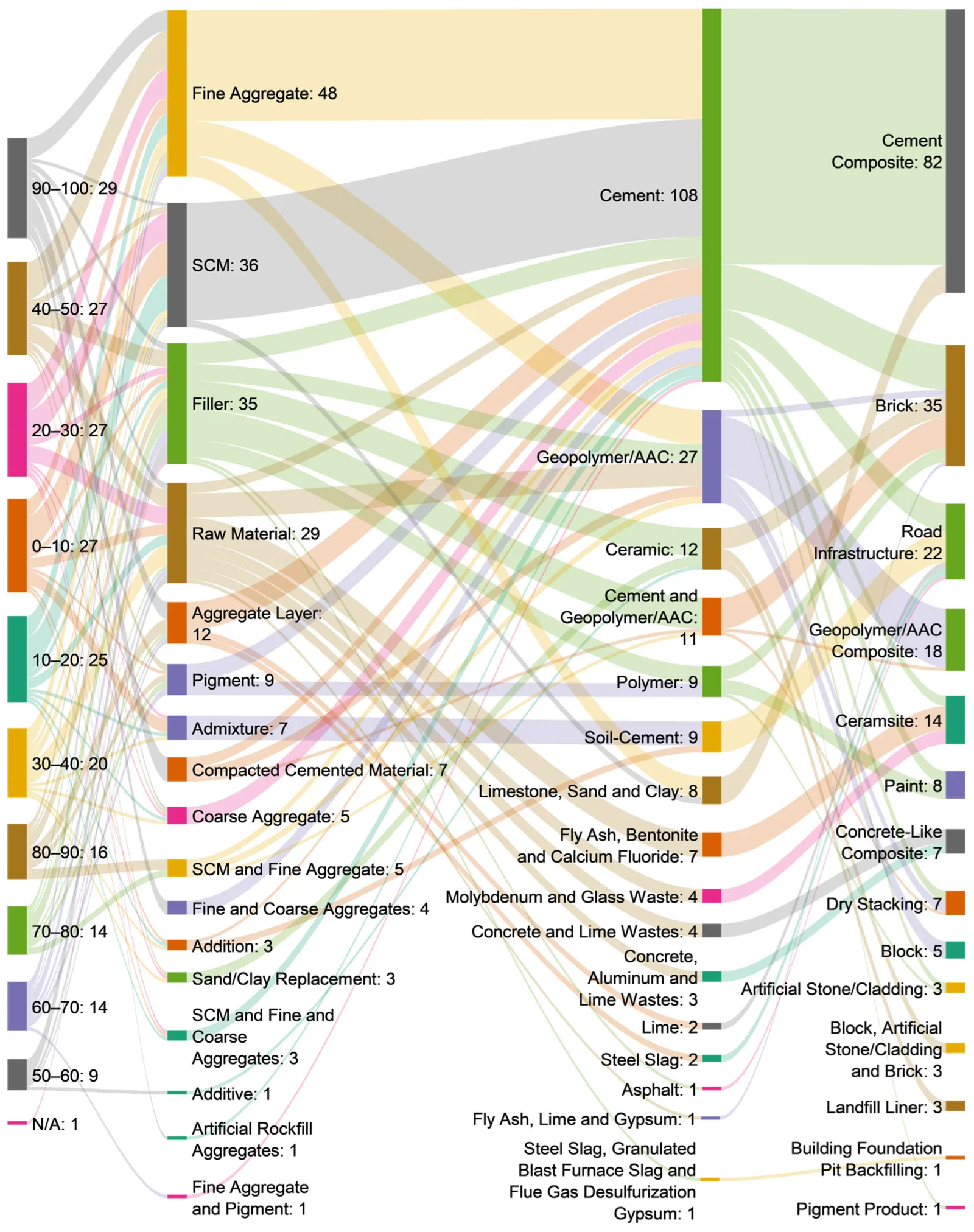
Flow of iron ore tailings from content to application in construction materials.

The composition of building materials incorporating IOT was analyzed and is presented in Figure 4. It is important to note that the total number of composites exceeds the number of articles, as many studies investigated multiple material proportions, compositions, and/or production processes within a single publication.

The most frequently used IOT incorporation range among researchers is 90–100%, primarily as fine aggregate (total replacement) in cement or geopolymer/alkali-activated cement (AAC)-based materials, in aggregate layers for road infrastructure, and as compacted cemented material for dry stacking applications. Lower incorporation ranges (0–50%) are more commonly applied as fine aggregate (partial replacement) or as supplementary cementitious materials (SCMs) in cement-based matrices, or as filler in various types of composites.

Specifically, IOT is most used as fine aggregate, particularly at contents of 40–50%, 20–30%, and 90–100%. When used as an SCM, IOT is typically incorporated at lower levels, especially within the 0–10%, 10–20%, and 20–30% ranges. The filler and raw material applications are also notable, spanning a broad range of IOT contents. It is worth noting that, although some authors classify fillers as SCM, this study does not adopt that classification. This decision is based on the use of non-cementitious matrices, in which IOT may function as a filler without contributing to cement hydration or pozzolanic reactions.

Among the matrices analyzed, Portland cement stands out as the most widely used, more so than all other matrices combined. This is particularly evident in the production of cement composites, bricks, and road infrastructure materials. This trend is expected, given cement’s dominant role in construction and its presence in concrete—the world’s second most consumed material after water [24].

Additionally, cement-based materials are a major focus of sustainability research due to their high environmental impact. Within these systems, IOT is predominantly used as fine aggregate in higher proportions or as SCM in smaller amounts (typically up to 30%, with 0–10% being most common). This is due to the potential negative effects of IOT on pozzolanic reactions, particularly when used as SCM in higher dosages [25]. Using IOT as SCM can help reduce CO_2_ emissions by partially replacing Portland cement, although often with lower technical performance and lower waste incorporation.

On the other hand, using IOT as an aggregate may lead to better technical properties and allow for higher waste incorporation, thus contributing more significantly to waste reduction and mitigating related environmental impacts. However, in this case, the CO_2_ emissions associated with Portland cement production remain unchanged. Therefore, the choice of how IOT is applied is closely linked to the researcher’s objectives, requiring a balance between technical performance and the environmental priorities [26].

The IOT is also employed in geopolymer matrices, mainly as fine aggregate or raw material, to produce geopolymer/AAC composites and blocks. As a filler, it appears in ceramic matrices for blocks, bricks, and artificial stone/cladding, in cement and geopolymer/AAC matrices for bricks, and in polymer matrices for bricks, paints, and artificial stones. Additionally, soil–cement matrices incorporating IOT are used in road infrastructure applications. Other alternative binders, primarily waste-based, have been explored in a smaller number of studies to produce diverse construction materials.

The overview of the composition of IOT-based building materials also reveals important research gaps. Notably, over the past decade, no studies have been identified that explore the use of hybrid matrices, such as polymer–geopolymer/AAC or polymer–cement systems. These combinations may offer synergistic benefits and warrant further investigation. Another promising direction for future research is the development of 3D-printed building materials, particularly those based on polymer matrices. This approach offers advantages such as design flexibility, potential for large-scale automated production, and reduced environmental impact through minimized material waste [27]. Lastly, only a limited number of studies were identified focusing on the use of IOT in the production of artificial stones and building foundation pit backfilling materials, highlighting another opportunity for future research.

### 3.3. Water Absorption of Iron Ore Tailing-Based Building Materials

#### 3.3.1. Effects of Iron Ore Tailing Contents in Cement and Geopolymer/AAC Matrices

**Figure 5:**
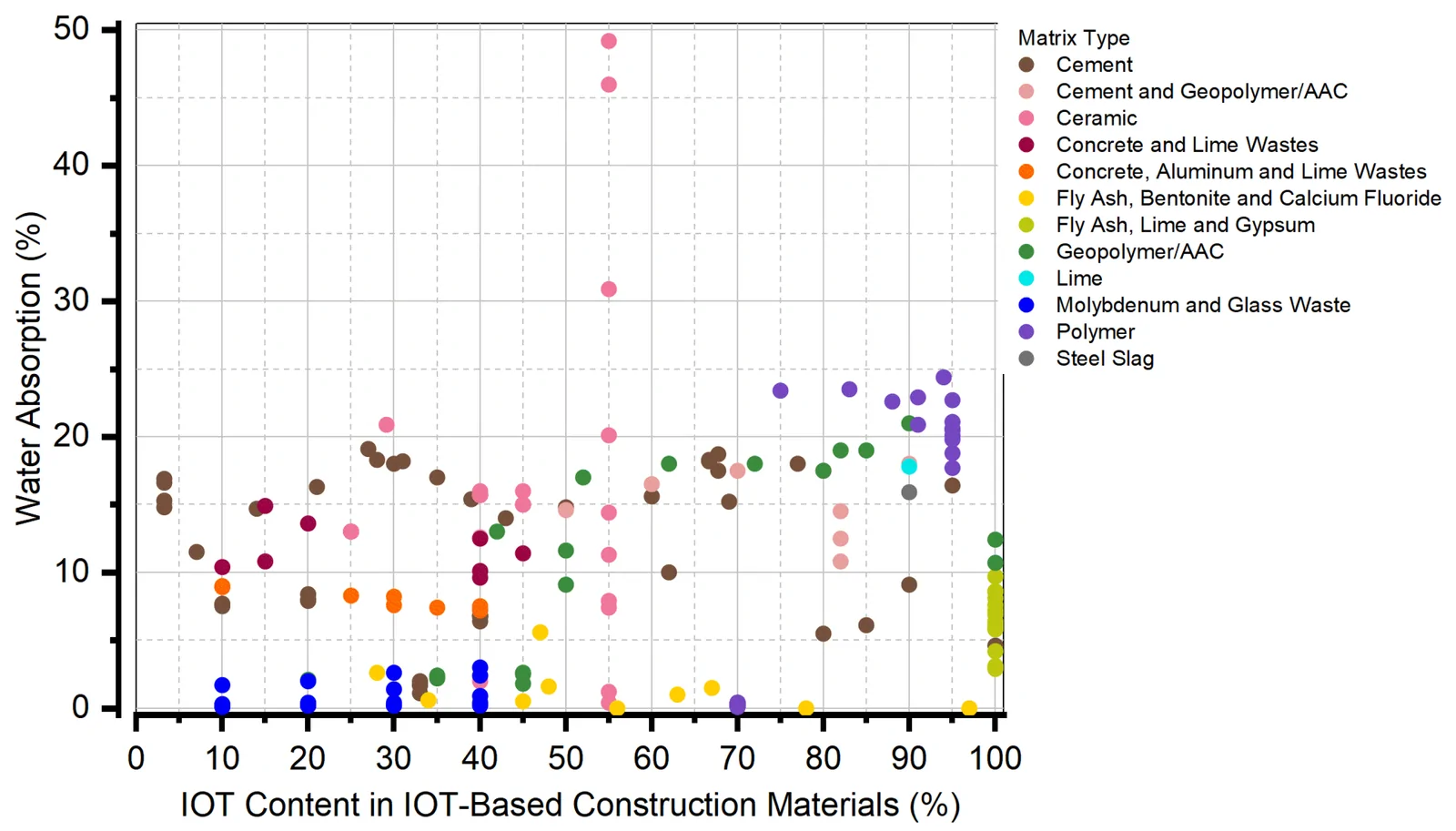
Water absorption of IOT-based building materials by IOT content and matrix type.

The water absorption performance of building materials incorporating IOT, categorized by IOT content and matrix type (data available on Supplementary Table S2) are presented in Figure 5 and Table 1. Individual quality assessment scores for each of the 24 studies are presented in Supplementary Table S3, with 54.2% classified as low risk and 45.8% as moderate risk (no study classified as high risk).

Correlation analysis (Table S6) revealed substantial heterogeneity in the strength and direction of the relationship between IOT content and water absorption across matrix types. The strongest associations were observed for polymer matrices (Pearson r = 0.90, Spearman ρ = 0.69, *p* < 0.001) and geopolymer/AAC systems (r = 0.70, ρ = 0.73, *p* ≤ 0.001), both showing a significant positive relationship. Conversely, concrete composites incorporating aluminum and lime wastes showed a strong negative association (r = −0.96, ρ = −0.94, *p* < 0.001), suggesting that in these systems, higher IOT content was linked to reduced porosity. For most other categories, correlations were weak and not statistically significant, indicating that IOT content alone is not a strong universal predictor of water absorption. Other factors, such as IOT particle size, firing/curing temperature, and processing conditions, likely moderate this relationship and were not captured in this cross-study analysis.

Although not all correlations were statistically significant, the negative influence of IOT content on water absorption in materials using Portland cement and geopolymer/AAC matrices, particularly in blocks, bricks, and cement composites, was due to three main mechanisms. First, IOT can hinder pozzolanic reactions, reducing the formation of hydration products and increasing porosity, especially when used as an SCM, which directly reduces cement content [28,29]. Second, the irregular shape of IOT particles leads to poor particle packing and a more porous microstructure [30,31]. Third, the fine particle size and high surface area of IOT increase water demand in the mixture, elevating the water-to-cement ratio and leaving more capillary pores after hydration [32].

The deterioration of microstructure in materials incorporating IOT into cement and/or AAC compromises their durability, primarily due to the high sensitivity of hydration and pozzolanic reactions [33]. Poor particle packing can lead to increased total porosity and the formation of larger pores [34,35]. These pores not only reduce mechanical strength by acting as stress concentrators but also facilitate the ingress of aggressive agents such as chlorides, sulfates, and carbon dioxide [36,37]. As a result, the material becomes more susceptible to degradation mechanisms including chloride corrosion, sulfate attack, and carbonation. Furthermore, insufficient pozzolanic activity or incomplete hydration can result in a more porous microstructure with weaker bonding, which also enables deeper penetration of fluids [38,39]. This type of porosity increases susceptibility to leaching and expansion-related damage. In particular, freeze–thaw cycles and alkali–silica reactions may occur, where water freezing or expansive gel formation within the pores generates internal pressures, leading to cracking, deformation, and long-term structural deterioration [40].

Eugênio et al. [25] developed a fiber–cement composite incorporating Portland cement, eucalyptus cellulose pulp, additives (hydroxypropylmethylcellulose—HPMC—and carboxylic polyester), and IOT as SCM. The water absorption values ranged from 11.5–18.3%, demonstrating a clear increase with higher IOT content. This behavior was attributed to the hindering of pozzolanic reactions and delayed cement hydration, which resulted in a more porous microstructure. Although water absorption increased with higher IOT content, some formulations achieved lower absorption than the reference sample (17.6%). This was attributed to the high fineness of IOT and its limited interference with pozzolanic reactions at lower incorporation levels, resulting in a denser microstructure. Thus, IOT incorporation helped preserve the cellulose fibers by reducing their exposure to the high alkalinity of the cement matrix, partially mitigating fiber degradation. As a result, the composites achieved flexural strength (modulus of rupture) values of 9.82–5.39 MPa, showing an inverse correlation with both IOT content and water absorption.

In another study, the same authors [28] produced blocks composed of lime, ground granulated blast furnace slag and IOT in contents ranging from 42% to 82%. Water absorption increased from 13% to 19% with higher IOT content. However, higher ground granulated blast furnace slag content counterbalanced the negative effect of IOT in microstructure by accelerating dissolution of amorphous silica from both IOT and ground granulated blast furnace slag, enhancing the formation of pozzolanic reaction products. Still, the block with the highest IOT content exhibited visible efflorescence, attributed to its porous microstructure, which facilitates the migration of soluble salts toward the surface.

Kakodkar and Sawaiker [29] developed blocks using a cement-based matrix incorporating ground granulated blast furnace slag, fly ash, lime, and total replacement of fine aggregate with IOT. The resulting composites exhibited an average water absorption of 4.6%. The authors observed that increasing the content of ground granulated blast furnace slag and fly ash contributed to reduced water absorption through chemical reactions between calcium and the silica-alumina phases present in the IOT, which enhanced bonding. Furthermore, the presence of fine particles from these additions helped refine the pore structure, resulting in a denser microstructure. Consequently, the blocks achieved compressive strengths ranging from 7.92 to 8.53 MPa after 28 days of curing. Regarding durability, the authors did not observe efflorescence on the cement-based blocks, being due to the high alumina content promoted by the ground granulated blast furnace slag and fly ash, resulting in stronger matrix bonding and limited alkali mobility.

In another study, Borges et al. [30] produced geopolymer/AAC composites using IOT sourced from jig concentrator and/or spiral classifier as partial (50%) and total (100%) replacements for fine aggregate. Water absorption increased with both IOT content (from 50% to 100%) and the solid-to-binder (s/b) ratio (from 1.2 to 1.3), reaching values of 9.1–10.7% with 50 wt% IOT and 11.6–12.4% for 100 wt% IOT, by increasing s/b from 1.2 to 1.3, respectively. This increase in water absorption was primarily attributed to higher porosity and larger average pore size, resulting from reduced particle packing efficiency. The effect was especially pronounced when IOT from the jig concentrator was used, due to its greater particle irregularity. As a result, the composites exhibited compressive strengths ranging from 36.3 to 49.5 MPa for 1.2 s/b ratio and 36.9 to 46.25 MPa for 1.3 s/b ratio.

#### 3.3.2. Effects of Iron Ore Tailing Contents on Polymer Matrices

**Figure 6:**
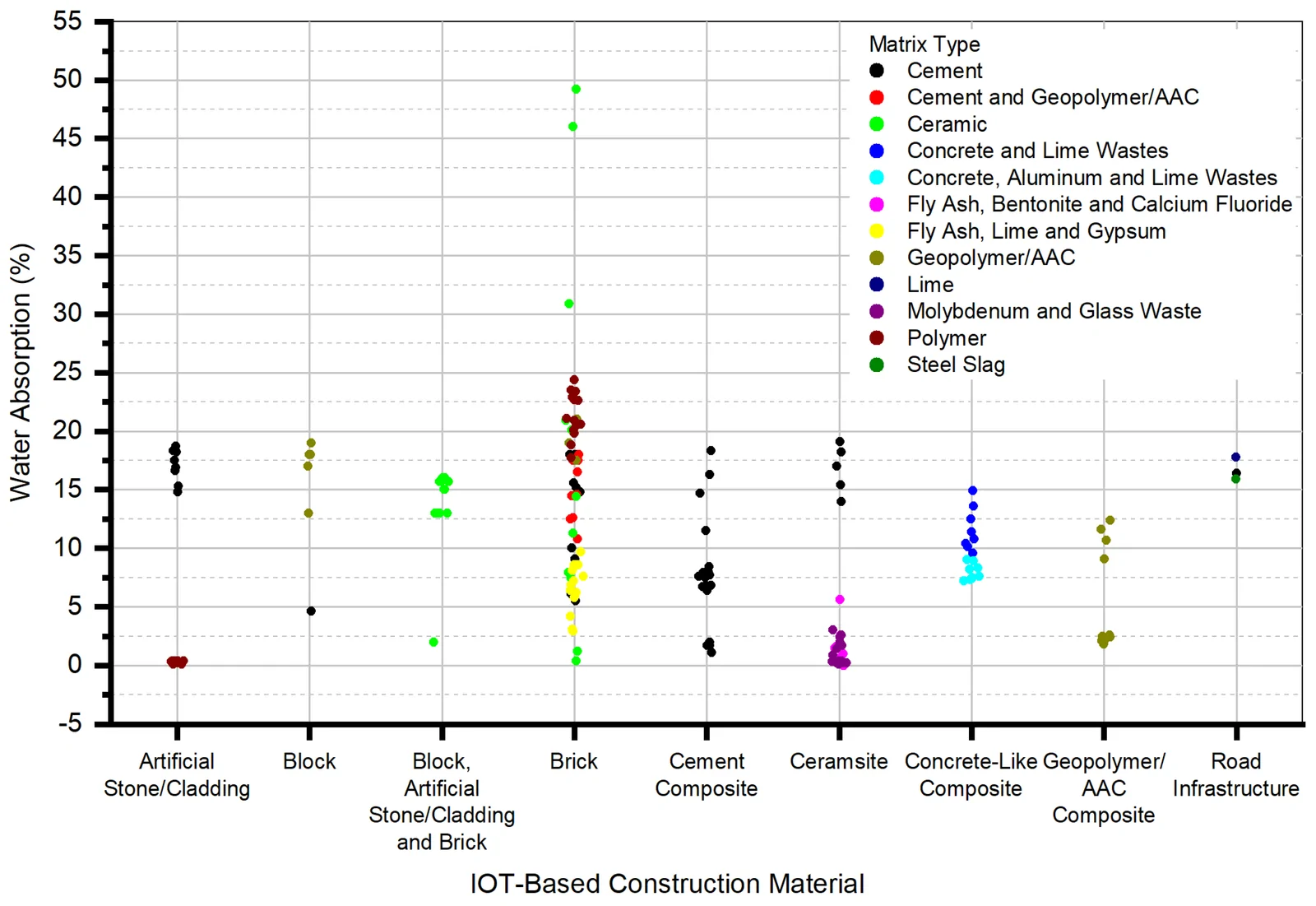
Water absorption of IOT-based building materials by IOT-based building material and matrix type.

Through Figure 6, water absorption can be analyzed according to type of IOT-based construction material and matrix type. IOT-based building materials incorporating polymer matrices can offer enhanced durability, largely due to the wide range of available polymers, which allows for tailored selection of the matrix according to the desired final properties. Once fully cured, polymer composites typically exhibit low water absorption, which highly contributes to their long-term durability by limiting the ingress of aggressive agents [41].

However, water uptake during the pre-curing phase can induce a plasticizing effect, increasing molecular chain mobility and consequently reducing both mechanical strength and durability [42]. In addition, many polymers, particularly thermoplastics, are inherently less resistant to thermal degradation and abrasive wear due to their relatively low thermal stability and surface hardness compared to inorganic matrices [43]. The incorporation of IOT as a mineral filler helps address these limitations, improving thermal resistance and hardness while further refining the microstructure, thus enhancing the mechanical performance and durability of the final composite [44,45].

The type of matrix used strongly influences water absorption of IOT-based artificial ornamental stone (AOS), where despite minimal variation in IOT content, substantial differences in water absorption are observed. Polymer matrices tend to produce materials with lower water absorption due to their ability to form a more continuous and denser microstructure. Additionally, polymers are primarily composed of nonpolar carbon chains, which contribute to their hydrophobic nature and reduced water uptake.

This contrast is evident when comparing the studies by Fontes et al. [32] and Silva and Paiva [46]. Fontes et al. produced hydraulic tiles using a Portland cement matrix with IOT incorporated as pigment and/or fine aggregate, while Silva and Paiva developed AOS, also intended for cladding applications, using polyester and epoxy polymer matrices with 70 wt% IOT as a filler. In the cement-based hydraulic tiles, water absorption ranged from 14.8% to 18.7% with up to approximately 67 wt% IOT. The study reported an increase in water demand, and consequently in the water-to-cement ratio, attributed to the high fineness and irregular morphology of IOT particles, characterized by angular shapes and sharp edges, leading to a higher porosity. In contrast, the polymer-based AOS exhibited significantly lower water absorption values, 0.14% to 0.44% for the polyester matrix and 0.14% to 0.20% for the epoxy matrix, with IOT contents as high as 70 wt%. As a result, despite the inherently brittle nature of cementitious materials, the AOS developed by Silva and Paiva achieved substantially higher flexural strengths (20.4–43.1 MPa for polyester and 27.5–46.2 MPa for epoxy) compared to the hydraulic tiles by Fontes et al. (4.77–12.18 MPa), even with similar levels of IOT incorporation.

In addition, the work by Silva and Paiva [41] showed that the epoxy-based composites demonstrated superior performance, including lower water absorption, compared to those made with polyester. This difference is attributed to the molecular structure of the polymers. Polyester contains ester groups (–COO–), which are polar and more prone to hydrolysis, making the material more susceptible to water absorption [47]. In contrast, epoxy resins form a highly cross-linked network of ether (–C–O–C–) and hydroxyl (–OH) groups through reactions with amine hardeners (R–NH_2_). This dense cross-linking restricts molecular chain mobility and limits the free volume available for water permeation, enhancing the material’s resistance to moisture. The results show that, in addition to the general matrix type, the specific material selected as the matrix also significantly influences the final properties of IOT-based building materials, especially the polymer-based matrices.

#### 3.3.3. Effects of Iron Ore Tailing Application Roles Through Different Matrices

When stratified by IOT functional role (Supplementary Table S5), the strongest and most significant association was observed when IOT was used as fine aggregate (Spearman ρ = 0.85, *p* < 0.001), indicating that water absorption increases consistently and strongly with IOT content in this role. A significant, moderate positive association was also found when IOT was used as a supplementary cementitious material (SCM) (ρ = 0.67, *p* = 0.012), and a weaker but still significant positive association was found when IOT was used as filler (ρ = 0.29, *p* = 0.036), despite the large sample size (*n* = 51) and considerable data dispersion in this category. In contrast, no significant association was found when IOT was combined with SCM and fine aggregate roles, used as raw material, or as sand/clay replacement, and no association at all was observed for the aggregate layer role (*n* = 3). Correlation could not be computed for the pigment, fine aggregate and pigment, and addition roles, as IOT content did not vary among the available data points in these categories. These results suggest that the functional role of IOT, particularly as fine aggregate, is a stronger and more consistent driver of water absorption than matrix type or product category alone, reinforcing the importance of considering IOT’s role in the mix design when predicting durability performance.

According to the authors [48] and complemented by Thejas and Hossiney [49], the use of IOT, particularly as an SCM, reduces the formation of hydration products, leading to weaker bonding and a less dense microstructure. These conditions increase susceptibility to efflorescence due to unreacted sodium oxide, which enhances moisture transport to the surface; and the presence of sodium silicate in geopolymer systems, which is particularly reactive with atmospheric humidity and CO_2_.

Additionally, Singh et al. [48] produced bricks made with Portland cement and/or geopolymer matrices, incorporating IOT as SCM and fine aggregate in contents ranging from 62% to 90 wt%. Geopolymer bricks with IOT as both SCM and fine aggregate exhibited the highest water absorption values (17.5–21.0%), followed by cement bricks with IOT as SCM only (10.0–18.0%), cement–geopolymer bricks with IOT as SCM and fine aggregate (10.8–14.5%), and cement bricks with IOT as both SCM and fine aggregate (5.5–9.0%). Slight efflorescence was noted in the cement–geopolymer bricks, whereas moderate to severe efflorescence occurred in geopolymer bricks, which was due to the susceptibility of sodium silicate to react with atmospheric moisture and CO_2_.

#### 3.3.4. Effects of Iron Ore Tailing Through Different Products and Production Methods

The effects of IOT on water absorption vary depending on the product type (Figure 6), likely due to differences in production processes. Correlation analysis (Table S7) revealed substantial heterogeneity in the strength and direction of the relationship between IOT content and water absorption across product categories. Artificial stone/cladding, bricks and cement composite products showed a significant negative association (r = −0.57, −0.25 and 0.41, and ρ = −0.77, −0.28 and −0.59, respectively), suggesting that in these systems, higher IOT content was linked to reduced porosity. On the other hand, an inverse correlation was observed for geopolymer/AAC composites (r = 0.77 and ρ = 0.84). For most other categories, correlations were weak and not statistically significant.

In some cases, such as ceramsite (a lightweight, sintered ceramic aggregate produced by pelletizing and firing raw materials until they bloat, forming a porous, low-density structure used as an alternative to natural aggregate) and specific cement composites, IOT incorporation led to reduced water absorption. This was attributed to the filler effect, where fine IOT particles filled voids between coarser ones, enhancing packing density and reducing porosity [25,50,51]. For instance, Almada et al. [50] produced structural mortars using Portland cement, quartz sand, and various types of IOT as a 40 wt% addition. The incorporation of IOT led to a reduction in water absorption to approximately 6.4–6.7%, compared to 7.5% in the reference sample without IOT. This improvement was attributed to enhanced microstructural packing, as the fine IOT particles filled the pores. Zhong et al. [51] also reported reduced water absorption in ceramsite aggregates produced with Portland cement, coal gangue, fly ash, sodium hydroxide, aluminum powder, quicklime, desulfurized gypsum, and IOT at incorporation levels of 27–43 wt%. Water absorption decreased from approximately 19.1% at 27 wt% IOT to 14.0% at 43 wt% IOT, attributed to the pore-filling effect of fine IOT particles, which contributed to a more compact and refined internal structure.

Among the various factors influencing water absorption in IOT-based building materials, the production method is also considered a source of variation, even though the limited number of studies reporting comparable production conditions does not allow robust quantitative relationships to be established between specific parameters, such as sintering temperature or curing conditions, and water absorption. In their work, Li et al. [52] produced a ceramic material with feldspar, clay, quartz, silicon carbide (foaming agent), sodium phosphate and 55 wt% IOT at 1110 °C, 1130 °C and 1150 °C sintering temperatures and with 0.00–0.15 wt% of foaming agent contents. They observed water absorption values of 0.3%, 1.2–11.3%, 7.9–30.9% and 20.1–49.2% at 0.00, 0.05, 0.10 and 0.15 wt% of silicon carbide, respectively, with decreasing sintering temperature. Therefore, there was an increase in water absorption with increase in foaming agent content and decrease in sintering temperature. According to the authors, the foaming agent generates bubbles that become pores, increasing pores sizes and water absorption, whereas at higher contents and higher temperatures, the bubbles collapse and create open pores, also increasing water absorption. The increase in sintering temperature can eliminate the bubbles, especially at lower foaming agent contents, and results in a denser interior microstructure, with lower water absorption. As stated by the authors, the higher water absorption also decreases resistance to expansion and freeze–thaw cycles.

Zhang et al. [53] produced ceramsite from molybdenum ore tailings, waste glass powder, 0.3 wt% silicon carbide as foaming agent and 10–40 wt% IOT in 10 wt% intervals. 1000, 1110, 1120, 1130 and 1140 °C sintering temperatures were used, resulting in water absorptions of 0.1–0.2%, 0.2–0.4%, 0.2–0.9%, 0.3–2.4% and 1.7–3.0% for 10, 20, 30 and 40 wt% IOT, respectively. Therefore, there was a trend of increase in water absorption with increase in sintering temperature and IOT content, probably due to increase in bubble formation and, consequently, interior pores that were not eliminated by the high sintering temperatures.

Conversely, the bricks produced by Weishi et al. [54], composed of fly ash, lime, gypsum, IOT, and chemical admixtures, specifically triethanolamine (a hardening accelerator) and stearic acid emulsion (a waterproofing agent), exhibited decreasing water absorption with increasing waterproofing agent content and higher initial curing temperatures. For bricks cured at 60 °C with 0.0–0.5% stearic acid emulsion, water absorption fell in the range of 9.7–4.2%. The waterproofing agent was reported to adsorb onto particle surfaces, reducing water ingress. By increasing the curing temperature from 30 °C to 60 °C for bricks without stearic acid, absorption values were 8.6–5.8%, while with 0.3% stearic acid values dropped to 8.1–2.9%. The improved water resistance at higher temperatures was attributed to enhanced dissolution and distribution of stearic acid, which blocked pores more effectively. However, the agent could also hinder the formation of hydration products, weakening microstructure at lower curing temperatures. Higher temperatures can promote better hydration, resulting in denser structures and lower absorption.

In polymer-based materials, production parameters, particularly curing conditions, have a significant influence on final properties. For example, in the work by Silva and Paiva [41], the authors also evaluated the effects of vacuum vibration during fabrication in the properties of AOS with 70 wt% IOT using polyester and epoxy polymer matrices. For AOS produced without vacuum vibration, water absorption values ranged from 0.29–0.40% for the polyester matrix and 0.14–0.17% for the epoxy matrix. When vacuum vibration was applied, water absorption increased to 0.38–0.44% (polyester) and 0.14–0.20% (epoxy). The authors attributed this increase in water absorption to reduced adhesion between the IOT particles and the polymer matrix. This effect also impacted mechanical performance, where flexural strength values dropped from 33.0–43.1 MPa (without vibration) to 20.4–34.5 MPa (with vibration) for polyester-based AOS, and from 38.2–40.0 MPa (without vibration) to 27.7–46.2 MPa (with vibration) for epoxy-based AOS. These results highlight the importance of optimizing production methods to balance microstructural quality, water resistance, and mechanical performance in polymer-based composites.

#### 3.3.5. Effects of Iron Ore Tailing Properties in the Produced Construction/Building Materials

It is also essential to consider the characteristics of IOT itself, as they can present direct impacts on the final properties of the materials. As previously observed, particle size distribution and morphology influence the packing density and, consequently, the water/cement ratio in cement and geopolymer systems [29,30,32,50,51]. Furthermore, the literature reports differences in product performance associated with variations in IOT chemical composition, as chemical composition can affect the reactivity and performance of cementitious matrices [28,30,31]. To further examine this relationship, the chemical composition data reported across the reviewed studies were compiled (Table S8), and correlation analyses were performed between the contents of the main oxides and water absorption (Table S9).

In the fiber–cement composite made by Eugênio et al. [25], the authors also observed that the chemical composition of the IOT affected the material’s microstructure, where the high SiO_2_ content in IOT (79.60%) contributed to pozzolanic activity, enhancing the formation of hydration products. This resulted in a refined pore structure, with reductions in both pore size and volume. Thejas and Hossiney [28] reported similar effects in blocks made with lime, ground granulated blast furnace slag, and IOT. The presence of ground granulated blast furnace slag accelerated the dissolution of SiO_2_, also present in IOT (35.49%), enhancing pozzolanic reactions and increasing the formation of aluminosilicate compounds, thereby improving the microstructure.

On the other hand, Vescovi et al. [31] found statistically significant differences in water absorption performance in Portland cement composites made with different types of IOT used as SCM at 10 and 20 wt% replacement levels. The IOT with higher Fe_2_O_3_ content yielded the lowest water absorption values (7.52% and 7.93% for 10% and 20% replacement, respectively), followed by the natural IOT (7.55% and 8.39%), and the IOT with lower Fe_2_O_3_ content (7.71% and 7.97%).

Although SiO_2_ content itself did not show a statistically significant association with water absorption in the Spearman analysis (ρ = 0.03, *p* = 0.736), the SiO_2_/Fe_2_O_3_ and SiO_2_/Al_2_O_3_ ratios showed positive and statistically significant associations (ρ = 0.55 and 0.58, respectively; both *p* < 0.001). Fe_2_O_3_ content also showed a positive and statistically significant association with water absorption (ρ = 0.21, *p* = 0.008). In contrast, Al_2_O_3_, CaO, MgO, K_2_O, and Na_2_O showed negative and statistically significant associations, with ρ values of −0.16 (*p* = 0.042), −0.40 (*p* < 0.001), −0.57 (*p* < 0.001), −0.52 (*p* < 0.001), and −0.41 (*p* < 0.001), respectively.

These results suggest that not only the content but also the chemical composition of IOT is associated with water absorption and, consequently, microstructural development. However, it is important to note that the benefits of pozzolanic activity are typically limited to lower levels of IOT incorporation. Beyond a certain threshold, the reduction in Portland cement content can outweigh the benefits of IOT reactivity, leading to a net negative impact on hydration and pozzolanic reactions, and thus on the overall performance of the composite.

Overall, water absorption is a critical parameter for assessing the durability of IOT-based building materials, as it directly influences the permeability, mechanical integrity, and long-term durability to environmental degradation [55]. Higher water absorption typically correlates with increased porosity and microstructural flaws, which facilitate the ingress of aggressive agents and accelerate deterioration mechanisms such as corrosion, efflorescence, sulfate attack, carbonation, leaching, and freeze–thaw damage [56,57]. Conversely, lower water absorption, achieved through optimized production processes, polymer matrices, or the filler effect of finely graded IOT, enhances resistance to these damaging processes by limiting fluid penetration and reducing pore connectivity [50]. Thus, controlling water absorption through careful selection of matrix type, IOT characteristics, and processing conditions is essential for improving the performance and durability of these sustainable construction materials.

## 4. Limitations, Implications and Perspectives

Firstly, it is important to highlight that this review has some limitations. First, restricting the search to Scopus^®^ may have introduced a selection bias toward studies published in more visible, highly indexed, or higher-impact-factor journals, potentially underrepresenting relevant work published in regional or lower-impact-factor sources that address IOT valorization but are less indexed or harder to locate. Second, the subsample used for the detailed water absorption analysis (*n* = 24) is modest relative to the heterogeneity of testing conditions across studies. Third, the potential influence of methodological differences has been acknowledged as a limitation when interpreting cross-study comparisons and the relationship between IOT content and water absorption. Lastly, quality assessment using the bespoke checklist revealed that reporting of statistical variability was frequently incomplete (62.5% of studies), which limits confidence in direct numerical comparisons across studies and should be considered when interpreting the generalizations presented in this review.

Additionally, water absorption testing age varied considerably among the 24 studies analyzed in detail, ranging from 3 to 28 days, and was not reported at all in 41.7% of cases. Since water absorption in cementitious and geopolymeric systems typically decreases with curing age due to continued hydration and pore refinement, this unaccounted variability may confound the observed relationship between IOT content and water absorption, and should be considered when interpreting the correlation and trend analyses presented in this review.

Nonetheless, integrating IOT into construction materials offers notable environmental benefits from a life cycle perspective, such as lowering gas emissions, conserving natural resources, managing waste, and reducing disposal needs [29,58,59]. Partially replacing Portland cement or natural aggregates with IOT can potentially decrease CO_2_ emissions by about 6% to 44% per kilogram of material, depending on both the level of substitution and the matrix type selected [59,60,61]. Additionally, reusing IOT helps divert large amounts of waste from tailings dams and stockpiles, which lowers the risk of soil contamination, acid mine drainage, and damage to ecosystems [15,29]. This also reduces environmental and safety risks associated with potential failures of tailings dams [62].

An analysis of Brazilian IOT generation and trends in civil construction indicates that utilizing IOT as a substitute for raw materials may decrease the volume of IOT directed to tailings dams by up to 60% [63]. A significant advantage is the decreased demand for natural aggregates such as river sand and gravel. The extraction of these materials is frequently associated with riverbed degradation, reduced water quality, groundwater depletion, and conflicts over land use [64,65,66,67]. These findings underscore the dual benefits of IOT-based materials: reducing environmental impacts and supporting alignment with international SDGs, particularly SDG 9 (Industry, Innovation and Infrastructure), 11 (Sustainable Cities and Communities), 12 (Responsible Consumption and Production), and 13 (Climate Action) [23].

Economic aspects also emerge as a key factor in IOT valorization. Cost reductions of up to 35% in concrete production have been documented when IOT is utilized as a cement substitute, and savings of up to 40% have been achieved with its use as an aggregate substitute at levels up to 50% [15]. Similar results were achieved in another study, where a reduction of approximately 44% of total production cost was observed when 40% IOT was used as raw material [68]. This reduction is attributed to the lower cost of IOT when compared to conventional materials and the unneeded extraction of natural resources, in addition to the reuse of large volumes of IOT, minimizing costs associated with storage, maintenance and disposal [15,68]. Nevertheless, logistics plays a critical role in ensuring the economic feasibility of reusing IOT as a raw material in civil construction, since distribution generates logistical costs and, consequently, socio-environmental impacts [69].

A simulation comparing IOT transport via trucks versus conveyor belts revealed that both methods are economically feasible, as their per-tonne transportation costs remain below those of traditional materials, even when distances exceed 150 km. Road transport proved to be the most effective option, covering distances of up to 290 km while remaining within the prescribed budget. The study highlights that this reverse logistics approach is widely applicable because it uses standard mining equipment, helping to lower both the volume of tailings in dams and the related risks [70].

Despite these restrictions, iron ore mining and beneficiation operations continue to produce hundreds of millions of tonnes of IOT annually worldwide, particularly in major producing countries such as Brazil, China, Australia, and India [8,71]. However, the reuse rates continue to fall below 30%, indicating a potentially untapped market, particularly within emerging economies where infrastructure demand is substantial [71,72].

The reuse of IOT in construction materials offers significant benefits for public health by decreasing the volume of tailings deposited in dams. On a regional scale, this reduction is associated with mitigating the risk of catastrophic dam failures, which have historically led to fatalities, compromised water supply systems, and long-term adverse effects on the health of local communities [68]. Tailing dams are widely recognized as posing risks to the health and safety of nearby communities. Because of the unique mechanical properties of tailings and the way these dams operate, they are more likely to fail than traditional water reservoirs [73,74].

Although reusing IOT offers environmental and social advantages, it is crucial to carefully assess the presence of potentially toxic heavy metals before proceeding with its application [75]. These elements, if released into the environment or used in construction materials without appropriate oversight, may pose substantial risks to human health. Primary exposure pathways consist of groundwater leaching, inhalation of dust, and direct contact. These routes are especially significant in humid climates, where leaching occurs more readily [68,75,76].

At the industrial level, concerns primarily focus on occupational exposure during tailings management and processing. The presence of metals such as arsenic (As), cadmium (Cd), mercury (Hg), and lead (Pb) warrants particular attention, as these substances are toxic even at low concentrations and do not present beneficial biological functions in humans [76,77]. However, when IOT is incorporated into polymeric or cementitious matrices, notable decreases in leaching potential and dust emissions are observed, suggesting that the majority of associated risks are confined to the production stage [41,78,79,80]. In this context, the adoption of appropriate pre-treatment procedures, utilization of personal protective equipment, and implementation of dust control measures are critical to ensuring the safe and sustainable application of these materials on a large scale [81,82,83].

IOT use in construction materials is also closely tied to regulations and public policies that encourage sustainable resource management. In Brazil, the Public Policy for Solid Residues (PNRS) has advanced reverse logistics and supported sustainability and the circular economy [70]. The PNRS sets forth principles for residue reduction, shared responsibility, and the economic valorization of waste, providing a legal framework to promote sustainable practices across all sectors, including mining [84].

Comparable regulations exist in other nations, including China and India, both of which continue to advance the consolidation of public policies aimed at efficient and sustainable residue management. In China, policy initiatives pertaining to waste management emphasize safety, minimization, resource recovery, and recycling, reflecting key principles of circular economy [85,86]. Solid residue management in India remains a significant environmental and social challenge. However, the implementation of policies formulated in collaboration with international organizations, such as the United Nations Environment Programme (UNEP), the United Nations Economic Commission for Europe (UNECE), and the World Bank, has contributed to strengthening these efforts [87]. The solid waste management policies enacted in 2016 reflect the nation’s ongoing commitment to prioritizing sustainability and addressing public health, environmental, and infrastructure challenges [88]. These initiatives demonstrate the viability of implementing these approaches on a large scale, particularly in emerging economies.

Political and institutional mechanisms play a strategic role in facilitating the adoption of IOT as a secondary raw material. The development of public policies aimed at its valorization, through economic incentives, subsidies, and targeted regulations, can help to overcome cost–benefit barriers that presently favor the use of conventional resources [63]. Furthermore, policies that support applied research, technological advancement, and the consolidation of technical standards are essential for expanding the adoption of IOT-based products and ensuring their acceptance in the market [89].

Governance is another critical element. Advancing transparency and accountability, through clear delineation of responsibilities and robust mechanisms for holding mining companies and management entities accountable, represents an essential step toward establishing sustainable and socially responsible practices [89]. From the perspective of Institutional Theory, these initiatives strengthen regulatory and normative influences on organizational conduct, encouraging mineral sector companies to implement green innovation strategies and environmental policies that are consistent with societal expectations and SDG [90]. In this context, effective policies can foster an innovative approach to secondary mining, positioning IOT as a strategic resource with significant potential for large-scale industrial and commercial applications [5,68]. Moreover, using and highlighting IOT-based materials in sustainable certifications can make construction materials more competitive and trustworthy by reinforcing their technical standards and environmental compliance, which may boost market demand [63].

## 5. Conclusions

This systematic literature review examined the current state of research on the use of IOT in building materials, with particular focus on water absorption as a key performance indicator. The analysis of 74 experimental studies revealed a growing global interest in the valorization of IOT, particularly in countries like China, Brazil, and India, where iron ore production, and, consequently, tailings generation, is substantial. The review showed that:

- IOT has been successfully incorporated into a wide variety of building materials, including bricks, blocks and artificial stones. Cement-based matrices remain the most explored, while IOT was most frequently used as fine aggregate (in 40–50%, 20–30%, 90–100% content ranges, respectively) or supplementary cementitious material (up to 30%).
- Water absorption in IOT-based building materials is influenced by multiple interrelated factors, with matrix type, IOT content and characteristics (chemical composition, particles size distribution and morphology), and production methods emerging as the most significant ones. In general, higher IOT contents tend to increase water absorption due to greater porosity, reduced particle packing efficiency, or hindered hydration and pozzolanic reactions. Such factors collectively compromise microstructural integrity and durability by facilitating the ingress of aggressive agents.
- However, under specific conditions, IOT can play a beneficial role. When acting as a filler, especially in formulations with finely ground particles, IOT can enhance packing density and reduce porosity. Likewise, optimized production techniques, such as high-temperature sintering, proper curing methodology, or the use of waterproofing additives, can mitigate water absorption.
- Among matrix types, polymer-based systems consistently exhibited the lowest water absorption values due to their dense, hydrophobic nature and limited pore connectivity. Moreover, epoxy matrices outperformed polyester due to their higher cross-linking density and lower susceptibility to hydrolysis.

These findings highlight the importance of proper matrix selection, processing conditions, and IOT characteristics to control water absorption and improve the long-term durability of sustainable construction materials. In addition, despite the significant advances, the review also identified gaps and opportunities for future research. Limited studies explored hybrid matrix systems, 3D printing applications, or underrepresented products such as artificial stones and foundation backfill materials. Overall, the findings reinforce the potential of IOT as a sustainable alternative in building materials, contributing to waste valorization, environmental preservation, and the advancement of circular economy principles in the construction sector.

## Figures and Tables

**Table 1:** Summary of water absorption of IOT-based building materials by product type, matrix type and IOT content.

Product	Matrix Type	IOT Content (%)	Water Absorption (%)
Artificial Stone/Cladding	Cement	3	14.8–16.9
		67–68	17.5–18.7
	Polymer	70	0.1–0.4
Block	Cement	100	4.6
	Geopolymer/AAC	42–82	13.0–19.0
Block, Artificial Stone/Cladding and Brick	Ceramic	25	13.0
		40–45	2.0–16.0
Brick	Cement	30	18.0
		50–60	14.8–15.6
		62–69	10.0–15.2
		77–80	5.5–18.0
		85–90	6.1–9.1
	Cement and Geopolymer/AAC	40–50	12.6–14.6
		60–70	16.5–17.5
		80–90	10.8–18.0
	Ceramic	29	20.9
		55	0.4–49.2
	Fly Ash, Lime and Gypsum	100	2.9–9.7
	Geopolymer/AAC	80–90	17.5–21.0
	Polymer	75	23.4
		83–88	22.6–23.5
		91–95	17.7–24.4
Cement Composite	Cement	7	11.5
		10–20	7.5–14.7
		21–28	16.3–18.3
		33–40	1.1–6.8
Ceramsite ^1^	Cement	27	19.1
		31–39	15.4–18.2
		43	14.0
	Fly Ash, Bentonite and Calcium Fluoride	28	2.6
		34	0.6
		45–48	0.5–5.6
		56	0.0
		63–67	1.0–1.5
		78	0.0
		97	0.0
	Molybdenum and Glass Waste	10–20	0.1–2.0
		30–40	0.2–3.0
Concrete-Like Composite	Concrete and Lime Wastes	10–20	10.4–14.9
		40–45	9.6–12.5
	Concrete, Aluminum and Lime Wastes	10	8.9–9.0
		25–30	7.6–8.3
		35–40	7.2–7.5
Geopolymer/AAC Composite	Geopolymer/AAC	20	2.1
		35	2.2–2.4
		45	1.8–2.6
		50	9.1–11.6
		100	10.7–12.4
Road Infrastructure	Cement	95	16.4
	Lime	90	17.8
	Steel Slag	90	15.9

Notes: ^1^ Ceramsite: a lightweight, sintered ceramic aggregate produced by pelletizing and firing raw materials until they bloat, forming a porous, low-density structure used as an alternative to natural aggregate.

## Data Availability

The original contributions presented in this study are included in the article/Supplementary Material. Further inquiries can be directed to the corresponding author.

## Supplementary Materials

The following supporting information can be downloaded at: https://www.mdpi.com/article/10.3390/ma19204385/s1, File S1: PRISMA 2020 checklist; Table S1: Literature by year, country and publication source; Table S2: Summary of the standards, curing age, compositions and water absorption performance of the investigated literature; Table S3: Risk of Bias Assessment of the investigated literature; Table S4: Repeatability assessment of water absorption data extracted via WebPlotDigitizer^®^; Table S5: Correlation and linear regression analysis between IOT content and water absorption, stratified by IOT functional role; Table S6: Correlation and linear regression analysis between IOT content and water absorption, stratified by matrix type; Table S7: Correlation and linear regression analysis between IOT content and water absorption, stratified by product category; Table S8: Synthesis of IOT chemical profile, content and water absorption; Table S9: Correlation and linear regression analysis between IOT chemical profile and water absorption. The complete reference list of the reviewed studies [25,28,29,30,31,32,46,48,49,50,51,52,53,54,91,92,93,94,95,96,97,98,99,100,101,102,103,104,105,106,107,108,109,110,111,112,113,114,115,116,117,118,119,120,121,122,123,124,125,126,127,128,129,130,131,132,133,134,135,136,137,138,139,140,141,142,143,144,145,146,147,148,149,150] is reported in the Supplementary Materials.

## Abbreviations

The following abbreviations are used in this manuscript:

AAC	Alkali-Activated Cement
AOS	Artificial Ornamental Stone
HPMC	Hydroxypropylmethylcellulose
IF	Impact Factor
IOT	Iron Ore Tailing
JCR	Journal Citation Report
PICO	Population, Intervention, Comparison, and Outcome
PNRS	Public Policy for Solid Residues
PRISMA	Preferred Reporting Items for Systematic Reviews and Meta-Analyses
s/b	Solid-to-Binder Ratio
SCMs	Supplementary Cementitious Materials
SDGs	Sustainable Development Goals
UNECE	United Nations Economic Commission for Europe
UNEP	United Nations Environment Programme
