# Supplementary material for: Iron Ore Tailings in Construction Materials: A Systematic Review of Composition and Water Absorption Performance
Source: Materials (Basel). 2026 Oct 19;19(20):4385. doi: 10.3390/ma19204385 (PMC13706708; doi:10.3390/ma19204385)
Supplement: Supplementary file 1 [file materials-19-04385-s001.zip › materials-4603114-File S1-PRISMA 2020 checklist.pdf]

PRISMA

PRISMA 2020 for Abstracts Checklist

Section and Topic	Item #	Checklist item	Reported (Yes/No)
**TITLE**			
Title	1	Identify the report as a systematic review.	Yes
**BACKGROUND**			
Objectives	2	Provide an explicit statement of the main objective(s) or question(s) the review addresses.	Yes
**METHODS**			
Eligibility criteria	3	Specify the inclusion and exclusion criteria for the review.	Yes
Information sources	4	Specify the information sources (e.g. databases, registers) used to identify studies and the date when each was last searched.	Yes
Risk of bias	5	Specify the methods used to assess risk of bias in the included studies.	Yes
Synthesis of results	6	Specify the methods used to present and synthesise results.	Yes
**RESULTS**			
Included studies	7	Give the total number of included studies and participants and summarise relevant characteristics of studies.	Yes
Synthesis of results	8	Present results for main outcomes, preferably indicating the number of included studies and participants for each. If meta-analysis was done, report the summary estimate and confidence/credible interval. If comparing groups, indicate the direction of the effect (i.e. which group is favoured).	Yes
**DISCUSSION**			
Limitations of evidence	9	Provide a brief summary of the limitations of the evidence included in the review (e.g. study risk of bias, inconsistency and imprecision).	Yes
Interpretation	10	Provide a general interpretation of the results and important implications.	Yes
**OTHER**			
Funding	11	Specify the primary source of funding for the review.	Yes
Registration	12	Provide the register name and registration number.	Not Registered

From: Page MJ, McKenzie JE, Bossuyt PM, Boutron I, Hoffmann TC, Mulrow CD, et al. The PRISMA 2020 statement: an updated guideline for reporting systematic reviews. BMJ 2021;372:n71. doi: 10.1136/bmj.n71. This work is licensed under CC BY 4.0. To view a copy of this license, visit https://creativecommons.org/licenses/by/4.0/

PRISMA 2020 Checklist

Section and Topic	Item #	Checklist item	Location where item is reported
**TITLE**			
Title	1	Identify the report as a systematic review.	Title
**ABSTRACT**			
Abstract	2	See the PRISMA 2020 for Abstracts checklist.	Abstract
**INTRODUCTION**			
Rationale	3	Describe the rationale for the review in the context of existing knowledge.	Introduction, para. 1-3
Objectives	4	Provide an explicit statement of the objective(s) or question(s) the review addresses.	Introduction, final paragraph
**METHODS**			
Eligibility criteria	5	Specify the inclusion and exclusion criteria for the review and how studies were grouped for the syntheses.	Methodology - Eligibility criteria
Information sources	6	Specify all databases, registers, websites, organisations, reference lists and other sources searched or consulted to identify studies. Specify the date when each source was last searched or consulted.	Methodology - Search strategy (single database: Scopus; search date: March 25, 2025). Explicitly acknowledged as a limitation in Section 4.
Search strategy	7	Present the full search strategies for all databases, registers and websites, including any filters and limits used.	Methodology, para. 2: full Boolean string reproduced as text ("IOT" OR "iron ore tailing") AND ("building" OR "construction") AND "material" AND NOT "internet of things", plus Figures 1 and 2
Selection process	8	Specify the methods used to decide whether a study met the inclusion criteria of the review, including how many reviewers screened each record and each report retrieved, whether they worked independently, and if applicable, details of automation tools used in the process.	Methodology - Selection process (single reviewer; no independent duplicate screening - noted as a limitation)
Data collection process	9	Specify the methods used to collect data from reports, including how many reviewers collected data from each report, whether they worked independently, any processes for obtaining or confirming data from study investigators, and if applicable, details of automation tools used in the process.	Methodology - Data collection process (single reviewer; no independent duplicate extraction - noted as a limitation)
Data items	10a	List and define all outcomes for which data were sought. Specify whether all results that were compatible with each outcome domain in each study were sought (e.g. for all measures, time points, analyses), and if not, the methods used to decide which results to collect.	Methodology, para. 1: "The following data items were extracted from each included study: water absorption values (primary outcome)..."
	10b	List and define all other variables for which data were sought (e.g. participant and intervention characteristics, funding sources). Describe any assumptions made about any missing or unclear information.	Methodology, para. 1: "...matrix type, IOT content, IOT functional role, country of the first author, publication year, journal, and journal impact factor (IF)"
Study risk of bias assessment	11	Specify the methods used to assess risk of bias in the included studies, including details of the tool(s) used, how many reviewers assessed each study and whether they worked independently, and if applicable, details of automation tools used in the process.	Methodology - Quality assessment (bespoke six-criterion checklist)
Effect measures	12	Specify for each outcome the effect measure(s) (e.g. risk ratio, mean difference) used in the synthesis or presentation of results.	No meta-analysis performed (narrative review)
Synthesis methods	13a	Describe the processes used to decide which studies were eligible for each synthesis (e.g. tabulating the study intervention characteristics and comparing	Methodology - Synthesis methods

Section and Topic	Item #	Checklist item	Location where item is reported
		against the planned groups for each synthesis (item #5)).	
	13b	Describe any methods required to prepare the data for presentation or synthesis, such as handling of missing summary statistics, or data conversions.	No data conversion required (narrative synthesis)
	13c	Describe any methods used to tabulate or visually display results of individual studies and syntheses.	Results - Table 1 and figures grouping studies by matrix type
	13d	Describe any methods used to synthesize results and provide a rationale for the choice(s). If meta-analysis was performed, describe the model(s), method(s) to identify the presence and extent of statistical heterogeneity, and software package(s) used.	Methodology - Synthesis methods (narrative synthesis by matrix type; no meta-analysis)
	13e	Describe any methods used to explore possible causes of heterogeneity among study results (e.g. subgroup analysis, meta-regression).	No meta-analysis performed
	13f	Describe any sensitivity analyses conducted to assess robustness of the synthesized results.	No meta-analysis performed
Reporting bias assessment	14	Describe any methods used to assess risk of bias due to missing results in a synthesis (arising from reporting biases).	Methodology - Reporting bias statement (no formal assessment conducted; acknowledged as limitation)
Certainty assessment	15	Describe any methods used to assess certainty (or confidence) in the body of evidence for an outcome.	No GRADE or equivalent certainty assessment performed for this type of review
**RESULTS**			
Study selection	16a	Describe the results of the search and selection process, from the number of records identified in the search to the number of studies included in the review, ideally using a flow diagram.	Methodology - Fig. 2 (PRISMA flow diagram); 145→79→74→24
	16b	Cite studies that might appear to meet the inclusion criteria, but which were excluded, and explain why they were excluded.	Methodology - exclusion criteria and reasons described
Study characteristics	17	Cite each included study and present its characteristics.	Results - Table 1
Risk of bias in studies	18	Present assessments of risk of bias for each included study.	Section 3.3.1 (in-text) and Supplementary Table S3 (individual quality assessment scores for all 24 studies)
Results of individual studies	19	For all outcomes, present, for each study: (a) summary statistics for each group (where appropriate) and (b) an effect estimate and its precision (e.g. confidence/credible interval), ideally using structured tables or plots.	Section 3.3.1-3.3.5: each subsection now opens with a synthesis/mechanistic paragraph followed by supporting individual-study evidence (restructured from the original sequential per-study narrative); Table 1 and Supplementary Table S2 provide structured per-study values
Results of syntheses	20a	For each synthesis, briefly summarise the characteristics and risk of bias among contributing studies.	Section 3.3.1, para. 1: "54.2% classified as low risk and 45.8% as moderate risk" (also in Abstract)
	20b	Present results of all statistical syntheses conducted. If meta-analysis was done, present for each the summary estimate and its precision (e.g. confidence/credible interval) and measures of statistical heterogeneity. If comparing groups, describe the direction of the effect.	No meta-analysis performed
	20c	Present results of all investigations of possible causes of heterogeneity among study results.	No meta-analysis performed
	20d	Present results of all sensitivity analyses conducted to assess the robustness	No meta-analysis performed

PRISMA 2020 Checklist

Section and Topic	Item #	Checklist item	Location where item is reported
		of the synthesized results.	
Reporting biases	21	Present assessments of risk of bias due to missing results (arising from reporting biases) for each synthesis assessed.	Discussion / Limitations - reporting bias not formally assessed (acknowledged as limitation)
Certainty of evidence	22	Present assessments of certainty (or confidence) in the body of evidence for each outcome assessed.	No GRADE or equivalent certainty assessment performed
**DISCUSSION**			
Discussion	23a	Provide a general interpretation of the results in the context of other evidence.	Discussion / Results and discussion section
	23b	Discuss any limitations of the evidence included in the review.	Limitations section (heterogeneous testing methods; n=24 subsample; single-database search)
	23c	Discuss any limitations of the review processes used.	Limitations section
	23d	Discuss implications of the results for practice, policy, and future research.	Implications and Perspectives section
**OTHER INFORMATION**			
Registration and protocol	24a	Provide registration information for the review, including register name and registration number, or state that the review was not registered.	Methodology - Registration statement (review not registered)
	24b	Indicate where the review protocol can be accessed, or state that a protocol was not prepared.	Methodology - no protocol was prepared (implicit in 24a statement)
	24c	Describe and explain any amendments to information provided at registration or in the protocol.	Review was not registered; no protocol amendments to report
Support	25	Describe sources of financial or non-financial support for the review, and the role of the funders or sponsors in the review.	Funding section (CAPES)
Competing interests	26	Declare any competing interests of review authors.	Declaration of competing interest section
Availability of data, code and other materials	27	Report which of the following are publicly available and where they can be found: template data collection forms; data extracted from included studies; data used for all analyses; analytic code; any other materials used in the review.	Data availability section

From: Page MJ, McKenzie JE, Bossuyt PM, Boutron I, Hoffmann TC, Mulrow CD, et al. The PRISMA 2020 statement: an updated guideline for reporting systematic reviews. BMJ 2021;372:n71. doi: 10.1136/bmj.n71. This work is licensed under CC BY 4.0. To view a copy of this license, visit https://creativecommons.org/licenses/by/4.0/
