# Supplementary material for: Iron Ore Tailings in Construction Materials: A Systematic Review of Composition and Water Absorption Performance
Source: Materials (Basel). 2026 Oct 19;19(20):4385. doi: 10.3390/ma19204385 (PMC13706708; doi:10.3390/ma19204385)
Supplement: Supplementary file 1 [file materials-19-04385-s001.zip › materials-4603114-supplementary.pdf]

Table S1. Literature by year, country and publication source.

Ref.	Publication Year	Country (1st Author)	Journal	Impact Factor (IF)
[1]	2022	Brazil	Journal of Materials Research and Technology	6.2
[2]	2020	Nigeria	Infrastructures	2.7
[3]	2016	Brazil	Journal of Materials in Civil Engineering	3.1
[4]	2019	Brazil	REM - International Engineering Journal	0.1
[5]	2024	Brazil	Revista de Gestão Social e Ambiental	N/A
[6]	2017	Brazil	Construction and Building Materials	7.4
[7]	2023	Brazil	Indian Geotechnical Journal	1.4
[8]	2022	Brazil	Ceramics International	5.1
[9]	2020	India	Journal of Hazardous, Toxic, and Radioactive Waste	N/A
[10]	2024	Brazil	Journal of Materials in Civil Engineering	3.1
[11]	2024	Brazil	Transportation Geotechnics	4.9
[12]	2024	Morocco	Journal of Ecological Engineering	1.3
[13]	2017	Nigeria	Transportation Geotechnics	4.9
[14]	2024	Brazil	Construction and Building Materials	7.4
[15]	2024	Brazil	Canadian Geotechnical Journal	3.0
[16]	2021	Brazil	Journal of Sustainable Metallurgy	2.5
[17]	2016	Brazil	Construction and Building Materials	7.4
[18]	2018	Brazil	Clean Technologies and Environmental Policy	4.2
[19]	2018	Brazil	Journal of Cleaner Production	9.8
[20]	2022	China	Advances in Materials Science and Engineering	2.1
[21]	2021	China	Materials	3.1
[22]	2024	China	Environmental Science and Pollution Research	5.8
[23]	2024	Brazil	Journal of Materials in Civil Engineering	3.1
[24]	2022	China	Buildings	3.1
[25]	2024	China	Journal of Materials in Civil Engineering	3.1
[26]	2024	Iran	Construction and Building Materials	7.4
[27]	2023	China	Construction and Building Materials	7.4
[28]	2025	India	Nature Environment and Pollution Technology	N/A
[29]	2023	China	Buildings	3.1

Ref.	Publication Year	Country (1st Author)	Journal	Impact Factor (IF)
[30]	2025	Iran	Construction and Building Materials	7.4
[31]	2022	India	Innovative Infrastructure Solutions	2.3
[32]	2019	China	Construction and Building Materials	7.4
[33]	2024	China	Construction and Building Materials	7.4
[34]	2021	China	Journal of Material Cycles and Waste Management	2.7
[35]	2023	China	Construction and Building Materials	7.4
[36]	2024	China	Construction and Building Materials	7.4
[37]	2023	Brazil	Indian Geotechnical Journal	1.4
[38].	2022	Brazil	International Journal of Mining, Reclamation and Environment	N/A
[39]	2019	Brazil	Construction and Building Materials	7.4
[40]	2022	Brazil	The International Journal of Advanced Manufacturing Technology	N/A
[41]	2021	Brazil	Construction and Building Materials	7.4
[42]	2023	Nigeria	Journal of Hazardous, Toxic, and Radioactive Waste	N/A
[43]	2015	Nigeria	Transportation Geotechnics	4.9
[44]	2021	India	International Journal of Sustainable Engineering	3.6
[45]	2016	India	Current Science	1.1
[46]	2022	Brazil	Construction and Building Materials	7.4
[47]	2025	Brazil	Remediation Journal	N/A
[48]	2020	Brazil	Cerâmica	N/A
[49]	2021	India	Journal of Scientific & Industrial Research	0.7
[50]	2025	India	Transportation Infrastructure Geotechnology	2.6
[51]	2024	India	Journal of Building Engineering	6.7
[52]	2024	India	Environmental Engineering Research	3.0
[53]	2024	China	Journal of Environmental Management	8.0
[54]	2022	India	Case Studies in Construction Materials	6.5
[55]	2022	India	Cogent Engineering	2.1
[56]	2024	China	Alexandria Engineering Journal	6.2
[57]	2023	Brazil	Journal of Building Engineering	6.7
[58]	2024	China	Buildings	3.1

Ref.	Publication Year	Country (1st Author)	Journal	Impact Factor (IF)
[59]	2024	China	Construction and Building Materials	7.4
[60]	2025	China	Environmental Research	7.7
[61]	2024	China	Gels	5.0
[62]	2022	China	Journal of Cleaner Production	9.8
[63]	2018	China	Journal of Cleaner Production	9.8
[64]	2024	China	Case Studies in Construction Materials	6.5
[65]	2024	China	Developments in the Built Environment	6.2
[66]	2023	China	Journal of Building Engineering	6.7
[67]	2022	China	Construction and Building Materials	7.4
[68]	2022	China	Materials	3.1
[69]	2023	China	Materials Today Communications	3.7
[70]	2024	China	Construction and Building Materials	7.4
[71]	2023	China	Frontiers in Earth Science	2.0
[72]	2022	China	Structural Concrete	3.0
[73]	2024	China	Construction and Building Materials	7.4
[74]	2023	China	Buildings	3.1

Table S2. Summary of the standards, curing age, compositions and water absorption performance of the investigated literature.

Ref.	Standard	Curing Age (days)	IOT Content (%)	IOT Application	Matrix Type	Product	Water Absorption (%)
[1] [1]	ASTM C642-13 and BS EN 1015-18	28	40	Addition (IOT Type 1)	Cement	Cement Composite	6.7
				Addition (IOT Type 2)			6.8
				Addition (IOT Type 3)			6.8
				Addition (IOT Type 4)			6.4
[3] [1]	ABNT NBR 13555 - 2012	7	95	Aggregate Layer	Cement	Road Infrastructure	16.4*
			90		Lime		17.8*
					Steel Slag		15.9*
[4]	RILEM (1984) Vacuum Saturation	7	50	Fine Aggregate	Geopolymer/AAC (1.2 s/b ratio)	Geopolymer/AAC Composite	9.1
					Geopolymer/AAC (1.3 s/b ratio)		11.6
			100		Geopolymer/AAC (1.2 s/b ratio)		10.7
					Geopolymer/AAC (1.3 s/b ratio)		12.4
[8]	ABNT NBR 13818 - 1997	NI	25	Sand/Clay Replacement	Ceramic (Oven-Fired at 900 ºC)	Block, Artificial Stone/Cladding and Brick	13.0
					Ceramic (Oven-Fired at 950 ºC)		13.0
					Ceramic (Oven-Fired at 1000 ºC)		13.0
					Ceramic (Oven-Fired at 1050 ºC)		13.0
			40		Ceramic (Oven-Fired at 1200 ºC)		2.0
					Ceramic (Oven-Fired at 900 ºC)		15.7*
					Ceramic (Oven-Fired at 950 ºC)		15.7*
					Ceramic (Oven-Fired at 1050 ºC)		15.7*
					Ceramic (Oven-Fired at 1000 ºC)		16.0
			45		Ceramic (Oven-Fired at 900 ºC)		15.0
					Ceramic (Oven-Fired at 1000 ºC)		15.0
					Ceramic (Oven-Fired at 1050 ºC)		15.0
					Ceramic (Oven-Fired at 950 ºC)		16.0
[47]	ABNT NBR 8492 – 2012	NI	75	Filler	Polymer (9 MPa Compression and 3h Heating)	Brick	23.4
			83				23.5
			88				22.6
			91				20.9
					Polymer (6 MPa Compression and 8h Heating)		22.9
			94				24.4
			95		Polymer (9 MPa Compression and 3h Heating)		17.7
					Polymer (6 MPa Compression and 4h Heating)		18.8

Ref.	Standard	Curing Age (days)	IOT Content (%)	IOT Application	Matrix Type	Product	Water Absorption (%)
					Polymer (6 MPa Compression and 5h Heating)		19.8
					Polymer (6 MPa Compression and 7h Heating)		20.1
					Polymer (6 MPa Compression and 6h Heating)		20.5
					Polymer (6 MPa Compression and 3h Heating)		20.6
					Polymer (6 MPa Compression and 2h Heating)		21.1
					Polymer (6 MPa Compression and 8h Heating)		22.7
[14]	ASTM C948-01 and ABNT NBR 15498 - 2016	28	7	SCM	Cement	Cement Composite	11.5
			14				14.7
			21				16.3
			28				18.3
[16]	ABNT NBR 9778 - 2005	7	67	Fine Aggregate (IOT Type 1)	Cement	Artificial Stone/Cladding	18.3
				Fine Aggregate (IOT Type 2)			18.2
			68	Fine Aggregate (IOT Type 2) and Pigment (Type 2)			17.5
				Fine Aggregate (IOT Type 2) and Pigment (Type 4)			18.7
			3	Pigment (Type 1)			15.4
				Pigment (Type 2)			14.8
				Pigment (Type 3)			16.9
				Pigment (Type 4)			16.6
[54]	IS 3495: Part 1–4/2019	28	40	Filler	Cement and Geopolymer/AAC	Brick	12.6
			50				14.6
			60				16.5
			70				17.5
			80				17.5
			90				18.0
[28]	IS 2185-Part 1/2005	NI	100	Fine Aggregate	Cement	Block	4.6
[34]	Chinese test standard	NI	55	Filler (0.00% SiC)	Ceramic (1110, 1130 and 1150º Firing Temperatures)	Brick	0.4

Ref.	Standard	Curing Age (days)	IOT Content (%)	IOT Application	Matrix Type	Product	Water Absorption (%)
	(Boiling, non-specified)			Filler (0.05% SiC)	Ceramic (1150º Firing Temperature)		1.2
				Filler (0.05% SiC)	Ceramic (1130º Firing Temperature)		7.4
				Filler (0.10% SiC)	Ceramic (1150º Firing Temperature)		7.9
				Filler (0.05% SiC)	Ceramic (1110º Firing Temperature)		11.3
				Filler (0.10% SiC)	Ceramic (1130º Firing Temperature)		14.4
				Filler (0.15% SiC)	Ceramic (1150º Firing Temperature)		20.1
				Filler (0.10% SiC)	Ceramic (1110º Firing Temperature)		30.9
				Filler (0.15% SiC)	Ceramic (1130º Firing Temperature)		46.0
				Filler (0.15% SiC)	Ceramic (1110º Firing Temperature)		49.2
[39]	ABNT NBR 15270-2 - 2017	NI	29	Filler	Ceramic	Brick	20.9
[40]	CS GB/T 17431.1–2010	28	10	Raw Material	Concrete and Lime (25%) Wastes	Concrete-Like Composite	10.4
			15		Concrete and Lime (25%) Wastes		10.8
					Concrete and Lime (15%) Wastes		14.9
			20		Concrete and Lime (15%) Wastes		13.6
			40		Concrete and Lime (25%) Wastes		9.6
					Concrete and Lime (20%) Wastes		10.1
					Concrete and Lime (15%) Wastes		12.5
			45		Concrete and Lime (15%) Wastes		11.4
[41]	ABNT NBR 9778 - 2009	28	10	Raw Material	Concrete, Aluminum and Lime (25%) Wastes	Concrete-Like Composite	8.9
					Concrete, Aluminum and Lime (20%) Wastes		9.0
			25		Concrete, Aluminum and Lime (15%) Wastes		8.3
			30		Concrete, Aluminum and Lime (25%) Wastes		7.6
					Concrete, Aluminum and Lime (15%) Wastes		8.2
			35		Concrete, Aluminum and Lime (25%) Wastes		7.4
			40		Concrete, Aluminum and Lime (25%) Wastes		7.2
					Concrete, Aluminum and Lime (20%) Wastes		7.3

Ref.	Standard	Curing Age (days)	IOT Content (%)	IOT Application	Matrix Type	Product	Water Absorption (%)
					Concrete, Aluminum and Lime (15%) Wastes		7.5
[44]	Indian Standards (IS, non-specified)	NI	30	Filler	Cement	Brick	18.0
			50				14.8
			60				15.6
[48]	ASTM C373-88	7	70	Filler (Fine)	Polymer (Epoxy and Without Vacuum Vibration)	Artificial Stone/Cladding	0.1
				Filler (Fine)	Polymer (Epoxy and With Vacuum Vibration)		0.1
				Filler (Coarse)	Polymer (Epoxy and Without Vacuum Vibration)		0.2
				Filler (Medium)	Polymer (Epoxy and Without Vacuum Vibration)		0.2
				Filler (Coarse)	Polymer (Epoxy and With Vacuum Vibration)		0.2
				Filler (Medium)	Polymer (Epoxy and With Vacuum Vibration)		0.2
				Filler (Coarse)	Polymer (Polyester and Without Vacuum Vibration)		0.3
				Filler (Fine)	Polymer (Polyester and Without Vacuum Vibration)		0.3
				Filler (Fine)	Polymer (Polyester and With Vacuum Vibration)		0.4
				Filler (Medium)	Polymer (Polyester and Without Vacuum Vibration)		0.4
				Filler (Coarse)	Polymer (Polyester and With Vacuum Vibration)		0.4
				Filler (Medium)	Polymer (Polyester and With Vacuum Vibration)		0.4
[49] [1]	IS 3495 - Part 2/1992	NI	62	SCM	Cement	Brick	10.0
			69				15.2
			77				18.0
			80	SCM and Fine Aggregate			5.5
			85				6.1
			90				9.1

Ref.	Standard	Curing Age (days)	IOT Content (%)	IOT Application	Matrix Type	Product	Water Absorption (%)
			82		Cement (14%) and Geopolymer/AAC (4%)		10.8
			82		Cement (4%) and Geopolymer/AAC (14%)		12.5
			82		Cement (9%) and Geopolymer/AAC (9%)		14.5
			80		Geopolymer/AAC		17.5
			85				19.0
			90				21.0
[52] [1]	ASTM C642-1997	28	45	Fine Aggregate	Geopolymer/AAC (1.5% Steel Fiber, 60% BOFS)	Geopolymer/AAC Composite	1.8
					Geopolymer/AAC (1.5% Steel Fiber, 50% BOFS)		2.5
					(1.0% Steel Fiber, 70% BOFS)		2.6
			35		Geopolymer/AAC (1.5% Steel Fiber, 80% BOFS)		2.2
					Geopolymer/AAC (1.5% Steel Fiber, 0% BOFS)		2.4
			20		Geopolymer/AAC (0.8% Steel Fiber, 20% BOFS)		2.1
[55]	IS 3495 - Parts 1-4/1992	28	42	Filler	Geopolymer/AAC	Block	13.0
			52				17.0
			62				18.0
			72				18.0
			82				19.0
[57]	ABNT NBR 9778 - 2009	28	10	SCM (High Fe Content IOT)	Cement	Cement Composite	7.5
				SCM (Natural IOT)			7.6
				SCM (Low Fe Content IOT)			7.7
			20	SCM (High Fe Content IOT)			7.9
				SCM (Low Fe Content IOT)			8.0
				SCM (Natural IOT)			8.4
[59] [1]	CS GB/T 17431.1–2010	NI	28	Raw Material	Fly Ash, Bentonite and Calcium Fluoride	Ceramsite	2.6
			34				0.6
			45				0.5
			47				5.6
			48				1.6
			56				0.0

Ref.	Standard	Curing Age (days)	IOT Content (%)	IOT Application	Matrix Type	Product	Water Absorption (%)
			63				1.0
			67				1.5
			78				0.0
			97				0.0
[63] [1]	CS JC/T422-2007	NI	100	Raw Material (0.3% Stearic Acid Emulsion)	Fly Ash, Lime and Gypsum (60 ºC Cure)	Brick	2.9
				Raw Material (0.3% Stearic Acid Emulsion)	Fly Ash, Lime and Gypsum (50 ºC Cure)		3.1
				Raw Material (0.5% Stearic Acid Emulsion)	Fly Ash, Lime and Gypsum (60 ºC Cure)		4.2
				Raw Material (0.0% Stearic Acid Emulsion)	Fly Ash, Lime and Gypsum (60 ºC Cure)		5.8
				Raw Material (0.4% Stearic Acid Emulsion)	Fly Ash, Lime and Gypsum (60 ºC Cure)		5.9
				Raw Material (0.0% Stearic Acid Emulsion)	Fly Ash, Lime and Gypsum (50 ºC Cure)		6.2
				Raw Material (0.3% Stearic Acid Emulsion)	Fly Ash, Lime and Gypsum (40 ºC Cure)		6.4
				Raw Material (0.3% Stearic Acid Emulsion)	Fly Ash, Lime and Gypsum (60 ºC Cure)		6.9
				Raw Material (0.2% Stearic Acid Emulsion)	Fly Ash, Lime and Gypsum (60 ºC Cure)		7.2
				Raw Material (0.0% Stearic Acid Emulsion)	Fly Ash, Lime and Gypsum (40 ºC Cure)		7.6
				Raw Material (0.3% Stearic Acid Emulsion)	Fly Ash, Lime and Gypsum (30 ºC Cure)		8.1
				Raw Material (0.1% Stearic Acid Emulsion)	Fly Ash, Lime and Gypsum (60 ºC Cure)		8.6
				Raw Material (0.0% Stearic Acid Emulsion)	Fly Ash, Lime and Gypsum (30 ºC Cure)		8.6
				Raw Material (0.0% Stearic Acid Emulsion)	Fly Ash, Lime and Gypsum (60 ºC Cure)		9.7
[64]	NI	28	33	Fine Aggregate (5% nickel-iron allow powder)	Cement	Cement Composite	1.1
				Fine Aggregate (0% nickel-iron allow powder)			1.7

Ref.	Standard	Curing Age (days)	IOT Content (%)	IOT Application	Matrix Type	Product	Water Absorption (%)
				Fine Aggregate (1% nickel-iron allow powder)			1.7
				Fine Aggregate (3% nickel-iron allow powder)			2.0
[70]	CS GB/T 17431.2–1010	NI	10	Raw Material	Molybdenum and Glass Waste (1100 ºC Sintering)	Ceramsite	0.1
					Molybdenum and Glass Waste (1110 ºC Sintering)		0.2
					Molybdenum and Glass Waste (1120 ºC Sintering)		0.2
					Molybdenum and Glass Waste (1130 ºC Sintering)		0.3
					Molybdenum and Glass Waste (1140 ºC Sintering)		1.7
			20		Molybdenum and Glass Waste (1100 ºC Sintering)		0.2
					Molybdenum and Glass Waste (1110 ºC Sintering)		0.2
					Molybdenum and Glass Waste (1120 ºC Sintering)		0.3
					Molybdenum and Glass Waste (1130 ºC Sintering)		0.4
					Molybdenum and Glass Waste (1140 ºC Sintering)		2.0
			30		Molybdenum and Glass Waste (1100 ºC Sintering)		0.2
					Molybdenum and Glass Waste (1110 ºC Sintering)		0.2
					Molybdenum and Glass Waste (1120 ºC Sintering)		0.4
					Molybdenum and Glass Waste (1130 ºC Sintering)		1.4
					Molybdenum and Glass Waste (1140 ºC Sintering)		2.6
			40		Molybdenum and Glass Waste (1100 ºC Sintering)		0.2

Ref.	Standard	Curing Age (days)	IOT Content (%)	IOT Application	Matrix Type	Product	Water Absorption (%)
					Molybdenum and Glass Waste (1110 ºC Sintering)		0.4
					Molybdenum and Glass Waste (1120 ºC Sintering)		0.9
					Molybdenum and Glass Waste (1130 ºC Sintering)		2.4
					Molybdenum and Glass Waste (1140 ºC Sintering)		3.0
[73]	CS GB/T17431.2-2010	3	27	Raw Material	Cement	Ceramsite	19.1
			31				18.2
			35				17.0
			39				15.4
			43				14.0

Notes: * Mean of the presented values; [1] Data obtained through WebPlotDigitizer®; NI: Non-informed.

Table S3. Risk of Bias Assessment of the investigated literature.

Ref.	C1 - IOT characterization	C2 - Standardized test method	C3 - Mix composition reported	C4 - Replicates reported	C5 - Statistical variability reported	C6 - Funding/COI declared	Total score (0-6)	Risk classification	Reviewer notes
[1]	1	1	1	0	1	1	5	Low risk	-
[3]	1	1	1	0	1	0.5	4.5	Moderate risk	C6 - Funding source mentioned only within Acknowledgments (no dedicated Funding statement); no COI declaration present.
[4]	1	1	1	0.5	0	0.5	4	Moderate risk	C4 - N not stated explicitly for water absorption; inferred as up to 10 (broken halves reused from the 10 mechanical-test specimens).<br>C6 - Funding source mentioned only within Acknowledgments (no dedicated Funding statement); no COI declaration present.
[8]	1	1	1	1	0	1	5	Low risk	-
[47]	1	1	1	0.5	0.5	1	5	Low risk	C4 and C5 - N and SD reported for pilot-scale (n=10) and PET full-scale (n=5) results, but not for the final polyester full-scale condition (Test 22) used in the structural calculations.
[14]	1	1	1	1	1	1	6	Low risk	-

[16]	1	1	1	0	1	1	5	Low risk	-
[54]	1	1	1	0	1	1	5	Low risk	-
[28]	1	0.5	1	1	0	0.5	4	Moderate risk	C2 - Method described but no standard cited. C6 - Funding source mentioned only within Acknowledgments (no dedicated Funding statement); no COI declaration present.
[34]	1	0.5	1	0	1	1	4.5	Moderate risk	C2 - Method described but no standard cited.
[39]	1	1	1	1	0	1	5	Low risk	-
[40]	1	1	1	0.5	0	1	4.5	Moderate risk	C4 - n=55 samples/composition stated for the study overall (split across 9 curing ages: 3-720 days), but exact n for water absorption specifically at each age not clarified.
[41]	1	1	1	0.5	0	1	4.5	Moderate risk	C4 - n=600 total samples stated for the study overall (across compositions × 9 curing ages), but exact n for water absorption specifically at each age/composition not clarified.
[44]	0	0.5	1	1	0	1	3.5	Moderate risk	C2 - Water absorption tested "according to IS standard" but specific IS code number not cited in text.
[48]	1	1	1	1	1	0.5	5.5	Low risk	C6 - Funding source mentioned only within Acknowledgments (no dedicated Funding statement); no COI declaration present.
[49]	1	1	1	0	0	0	3	Moderate risk	-
[52]	1	1	1	1	0	1	5	Low risk	-
[55]	1	1	1	1	1	1	6	Low risk	-
[57]	1	1	1	1	1	1	6	Low risk	-
[59]	1	1	1	0	0	1	4	Moderate risk	-
[63]	1	1	1	0	0	1	4	Moderate risk	-
[64]	1	0.5	1	0	0	1	3.5	Moderate risk	C2 - Method described but no standard cited.
[70]	1	1	1	1	0	1	5	Low risk	-
[73]	1	1	1	1	1	1	6	Low risk	-

Notes:

IOT = iron ore tailings; SD = standard deviation; COI = conflict of interest; n = number of replicates/specimens

C1 = IOT characterization reported (chemical and/or physical); C2 = standardized, cited test method for water absorption; C3 = mix composition fully reported; C4 = number of replicates reported; C5 = statistical variability reported (standard deviation, coefficient of variation, or error bars); C6 = funding source or conflict-of-interest declared.

Each criterion was scored as 1 (Yes), 0.5 (Partial), or 0 (No). Total score = sum of C1–C6 (range 0–6).

Risk classification: Low risk (5–6); Moderate risk (3–4.5); High risk (0–2.5).

As no validated risk-of-bias tool exists for experimental studies in materials science and engineering, this bespoke six-criterion checklist was developed for this review and applied by a single reviewer to the 24 studies reporting water absorption data. Reviewer notes provide justification for partial (0.5) scores where applicable.

Table S4. Repeatability assessment of water absorption data extracted via WebPlotDigitizer®.

Study	Point	Measurement 1 (%)	Measurement 2 (%)	Measurement 3 (%)	Standard Deviation	Mean	Coefficient of Variation (%)
[1]	1	6.73	6.78	6.78	0.028868	6.76	0.426824
	2	6.82	6.82	6.82	0.000000	6.82	0.000000
	3	6.82	6.92	6.82	0.057735	6.85	0.842437
	4	6.44	6.39	6.44	0.028868	6.42	0.449416
[3]	1	16.40	16.35	16.40	0.025981	16.38	0.158613
	2	17.81	17.81	17.81	0.000000	17.81	0.000000
	3	15.84	15.82	15.84	0.011547	15.83	0.072928
[49]	1	18.08	18.08	18.09	0.005774	18.08	0.031927
	2	15.29	15.29	15.29	0.000000	15.29	0.000000
	3	10.16	10.16	10.16	0.000000	10.16	0.000000
	4	14.55	14.42	14.55	0.075056	14.51	0.517387
	5	12.48	12.36	12.48	0.069282	12.44	0.556930
	6	10.79	10.67	10.79	0.069282	10.75	0.644484
	7	21.04	20.92	20.92	0.069282	20.96	0.330544
	8	19.08	18.96	18.96	0.069282	19.00	0.364642
	9	17.46	17.46	17.46	0.000000	17.46	0.000000
	10	9.05	9.05	8.93	0.069282	9.01	0.768946
	11	5.92	6.04	6.04	0.069282	6.00	1.154701
	12	5.37	5.25	5.37	0.069282	5.33	1.299851
[52]	1	2.45	2.45	2.45	0.000000	2.45	0.000000
	2	2.10	2.10	2.10	0.000000	2.10	0.000000
	3	2.20	2.19	2.20	0.005774	2.20	0.262830
	4	2.60	2.60	2.60	0.000000	2.60	0.000000
	5	1.80	1.80	1.80	0.000000	1.80	0.000000
	6	2.50	2.51	2.51	0.005774	2.51	0.230326
[59]	1	2.54	2.53	2.56	0.015275	2.54	0.600600
	2	0.63	0.64	0.64	0.005774	0.64	0.906833
	3	0.49	0.50	0.51	0.010000	0.50	2.000000
	4	5.54	5.53	5.56	0.015275	5.54	0.275561
	5	1.60	1.60	1.64	0.023094	1.61	1.431447
	6	0.00	0.01	0.04	0.020817	0.02	124.899960
	7	1.03	1.03	1.04	0.005774	1.03	0.558726
	8	1.46	1.46	1.49	0.017321	1.47	1.178266

Study	Point	Measurement 1 (%)	Measurement 2 (%)	Measurement 3 (%)	Standard Deviation	Mean	Coefficient of Variation (%)
	9	0.00	0.00	0.01	0.005774	0.00	173.205081
	10	0.03	0.00	0.03	0.017321	0.02	86.602540
[63]	1	9.66	9.69	9.66	0.017321	9.67	0.179116
	2	8.62	8.62	8.62	0.000000	8.62	0.000000
	3	7.26	7.26	7.23	0.017321	7.25	0.238904
	4	6.86	6.89	6.89	0.017321	6.88	0.251752
	5	5.84	5.84	5.87	0.017321	5.85	0.296077
	6	4.19	4.17	4.17	0.011547	4.18	0.276465
	7	8.69	8.79	8.79	0.057735	8.76	0.659327
	8	7.66	7.73	7.73	0.040415	7.71	0.524410
	9	6.26	6.29	6.33	0.035119	6.29	0.558033
	10	5.83	5.83	5.90	0.040415	5.85	0.690453
	11	8.20	8.23	8.20	0.017321	8.21	0.210968
	12	6.58	6.51	6.51	0.040415	6.53	0.618590
	13	3.29	3.26	3.26	0.017321	3.27	0.529679
	14	3.10	3.10	3.10	0.000000	3.10	0.000000
				**Mean**	0.024415	-	8.261338
				**Median**	0.017321	-	0.364642
				**Maximum**	0.075056	-	173.205081

Table S5. Correlation and linear regression analysis between IOT content and water absorption, stratified by IOT functional role.

IOT Application	n	WA Min	WA Max	Pearson r	Pearson p-value	Spearman ρ	Spearman p-value	Slope	Intercept	R^2	Interpretation*
Pigment	42	1.1	19.1	0.01	0.936	-0.02	0.921	0.06	39.91	0.000	NA
Fine Aggregate and Pigment	9	10.8	18.0	0.18	0.642	0.06	0.879	1.19	52.90	0.033	NA
Fine Aggregate	24	0.4	49.2	0.17	0.417	0.01	0.947	0.17	41.40	0.030	Positive association, statistically significant
SCM and Fine Aggregate	8	9.6	14.9	-0.33	0.425	-0.17	0.684	-2.56	57.99	0.109	Positive association, not statistically significant
SCM	9	7.2	9.0	-0.96	< 0.001	-0.94	< 0.001	-16.62	160.76	0.922	Positive association, statistically significant
Filler	10	0.0	5.6	-0.42	0.225	-0.53	0.112	-5.09	63.12	0.178	Positive association, statistically significant
Addition	14	2.9	9.7	NA	NA	NA	NA	0.00	100.00	NA	NA

IOT Application	n	WA Min	WA Max	Pearson r	Pearson p-value	Spearman ρ	Spearman p-value	Slope	Intercept	R^2	Interpretation*
Raw Material	18	1.8	21.0	0.70	0.001	0.73	0.001	2.36	34.34	0.493	Positive association, not statistically significant
Aggregate Layer	1	17.8	17.8	NA	NA	NA	NA	NA	NA	NA	No association
Sand/Clay Replacement	20	0.1	3.0	0.36	0.119	0.42	0.067	4.34	21.25	0.129	Positive association, not statistically significant

Note:
n = number of data points; WA Min/WA Max = minimum/maximum water absorption values (%) observed within the category; r = Pearson correlation coefficient; ϱ = Spearman's rank correlation coefficient; p = two-tailed probability value; R^2 = coefficient of determination (r^2); NA = not applicable, assigned to categories with fewer than three data points or with no variance in IOT content among available data points, for which correlation and regression are not computable.
* Statistical significance (α = 0.05) was assessed using Spearman's ϱ, given the scattered, non-normally distributed nature of the water absorption data.

Table S6. Correlation and linear regression analysis between IOT content and water absorption, stratified by matrix type.

Matrix Type	n	WA Min	WA Max	Pearson r	Pearson p-value	Spearman ρ	Spearman p-value	Slope	Intercept	R^2	Interpretation*
Cement	42	1.1	19.1	0.01	0.936	-0.02	0.921	0.06	39.91	0.000	Negative association, not statistically significant
Cement and Geopolymer/AAC	9	10.8	18.0	0.18	0.642	0.06	0.879	1.19	52.90	0.033	Positive association, not statistically significant
Ceramic	24	0.4	49.2	0.17	0.417	0.01	0.947	0.17	41.40	0.030	Positive association, not statistically significant
Concrete and Lime Wastes	8	9.6	14.9	-0.33	0.425	-0.17	0.684	-2.56	57.99	0.109	Negative association, not statistically significant
Concrete, Aluminum and Lime Wastes	9	7.2	9.0	-0.96	< 0.001	-0.94	< 0.001	-16.62	160.76	0.922	Negative association, statistically significant
Fly Ash, Bentonite and Calcium Fluoride	10	0.0	5.6	-0.42	0.225	-0.53	0.112	-5.09	63.12	0.178	Negative association, not statistically significant
Fly Ash, Lime and Gypsum	14	2.9	9.7	NA	NA	NA	NA	0.00	100.00	NA	NA
Geopolymer/AAC	18	1.8	21.0	0.70	0.001	0.73	0.001	2.36	34.34	0.493	Positive association, statistically significant
Lime	1	17.8	17.8	NA	NA	NA	NA	NA	NA	NA	NA

Matrix Type	n	WA Min	WA Max	Pearson r	Pearson p-value	Spearman ρ	Spearman p-value	Slope	Intercept	R^2	Interpretation*
Molybdenum and Glass Waste	20	0.1	3.0	0.36	0.119	0.42	0.067	4.34	21.25	0.129	Positive association, not statistically significant
Polymer	26	0.1	24.4	0.90	< 0.001	0.69	< 0.001	0.98	70.26	0.805	Positive association, statistically significant
Steel Slag	1	15.9	15.9	NA	NA	NA	NA	NA	NA	NA	NA

Note:
n = number of data points; WA Min/WA Max = minimum/maximum water absorption values (%) observed within the category; r = Pearson correlation coefficient; ϱ = Spearman's rank correlation coefficient; p = two-tailed probability value; R^2 = coefficient of determination (r^2); NA = not applicable, assigned to categories with fewer than three data points or with no variance in IOT content among available data points, for which correlation and regression are not computable.
* Statistical significance (α = 0.05) was assessed using Spearman's ϱ, given the scattered, non-normally distributed nature of the water absorption data.

Table S7. Correlation and linear regression analysis between IOT content and water absorption, stratified by product category.

Product	n	WA Min	WA Max	Pearson r	Pearson p-value	Spearman ρ	Spearman p-value	Slope	Intercept	R^2	Interpretation*
Artificial Stone/Cladding	20	0.1	18.7	-0.57	0.008	-0.77	< 0.001	-1.84	68.91	0.330	Negative association, statistically significant
Block	6	4.6	19.0	-0.46	0.363	0.12	0.827	-1.75	94.46	0.209	Positive association, not statistically significant
Brick	60	0.4	49.2	-0.25	0.057	-0.28	0.033	-0.54	87.46	0.061	Negative association, statistically significant
Cement Composite	18	1.1	18.3	-0.41	0.087	-0.59	0.011	-1.01	33.01	0.172	Negative association, statistically significant
Ceramsite	35	0.0	19.1	-0.01	0.949	0.10	0.585	-0.04	35.50	0.000	Positive association, not statistically significant
Road Infrastructure	3	15.9	17.8	-0.26	0.830	0.00	1.000	-0.77	104.58	0.070	No association
Block, Artificial Stone/Cladding and Brick	13	2.0	16.0	0.19	0.532	0.52	0.065	0.44	30.92	0.036	Positive association, not statistically significant
Concrete-Like Composite	17	7.2	14.9	-0.29	0.261	-0.24	0.345	-1.58	43.81	0.083	Negative association, not statistically significant
Geopolymer/AAC Composite	10	1.8	12.4	0.77	0.009	0.84	0.002	4.51	26.62	0.599	Positive association, statistically significant

Note:
n = number of data points; WA Min/WA Max = minimum/maximum water absorption values (%) observed within the category; r = Pearson correlation coefficient; ϱ = Spearman's rank correlation coefficient; p = two-tailed probability value; R^2 = coefficient of determination (r^2).

Product	n	WA Min	WA Max	Pearson r	Pearson p-value	Spearman ρ	Spearman p-value	Slope	Intercept	R^2	Interpretation*

* Statistical significance (α = 0.05) was assessed using Spearman's ϱ, given the scattered, non-normally distributed nature of the water absorption data.

Table S8. Synthesis of IOT chemical profile, content and water absorption.

Article	n	IOT Content (%)	Water Absorption (%)	IOT Chemical Composition (Oxides, %)									
				SiO_2	Al_2O_3	Fe_2O_3	CaO	MgO	K_2O	TiO_2	Na_2O	SO_3	LOI
[1]	1	40	6.4	54.10	0.43	44.80	0.06	-	0.05	-	0.43	-	0.11
[1]	1	40	6.7	10.60	3.04	81.21	0.06	-	0.02	0.08	-	-	4.94
[1]	1	40	6.8	18.30	1.83	74.20	0.03	-	0.03	0.08	-	-	4.22
[1]	1	40	6.8	49.80	0.51	49.61	0.03	-	0.01	0.08	-	-	0.62
[3]	3	90-95	15.9-17.8	20.40	11.20	68.05	-	-	0.20	-	-	-	-
[4]	4	50-100	9.1-12.4	NS	-	NS	-	-	-	-	-	-	-
[8]	13	25-45	2-16	58.60	3.20	35.40	0.10	0.10	0.20	-	0.10	-	2.19
[47]	14	75-95	17.7-24.4	-	-	-	-	-	-	-	-	-	-
[14]	4	7-28	11.5-18.3	-	-	-	-	-	-	-	-	-	-
[16]	3	3-67	14.8-18.3	58.10	5.00	36.30	0.20	-	0.10	-	-	-	2.40
[16]	2	3	16.6-16.9	20.00	18.80	57.30	0.30	-	0.30	-	-	-	9.40
[16]	3	67-68	17.5-18.7	79.20	3.10	16.80	0.10	-	0.10	-	-	-	1.10
[54]	6	40-90	12.6-18	30.37	12.43	46.33	3.88	1.68	0.25	3.96	-	-	1.10
[28]	1	100	4.6	17.90	5.80	63.09	0.02	0.14	-	-	-	-	11.66
[34]	10	55	0.4-49.2	37.59	9.27	7.70	11.48	7.83	0.97	9.70	1.78	-	-
[39]	1	29	20.9	63.09	7.17	25.79	0.20	0.12	0.32	0.28	-	-	2.28
[40]	8	10-45	9.6-14.9	41.90	6.30	48.10	0.20	-	0.20	-	-	-	3.10
[41]	9	10-40	7.2-9	41.90	6.30	48.10	0.20	-	0.20	-	-	-	3.08
[44]	3	30-60	14.8-18	-	-	-	-	-	-	-	-	-	-
[48]	4	70	0.1-0.4	42.70	5.70	50.10	-	-	-	-	-	1.20	-
[48]	4	70	0.2-0.4	46.00	5.30	45.30	-	-	-	-	-	2.40	-
[48]	4	70	0.2-0.4	76.90	3.20	19.60	-	-	-	-	-	-	-
[49]	12	62-90	5.5-21	49.00	23.22	24.76	0.23	0.53	0.30	1.15	0.04	0.58	0.04

Article	n	IOT Content (%)	Water Absorption (%)	IOT Chemical Composition (Oxides, %)									
				SiO_2	Al_2O_3	Fe_2O_3	CaO	MgO	K_2O	TiO_2	Na_2O	SO_3	LOI
[52]	6	20-45	1.8-2.6	24.90	23.90	32.80	0.20	0.60	1.03	0.40	-	0.01	-
[55]	5	42-82	13-19	35.49	8.89	40.19	3.30	1.24	0.25	7.14	-	-	3.50
[57]	2	10-20	7.5-7.9	17.69	3.38	68.42	-	-	-	-	-	0.17	-
[57]	2	10-20	7.6-8.4	27.11	2.20	56.59	-	-	-	-	-	0.17	-
[57]	2	10-20	7.7-8	35.78	3.38	45.99	-	-	-	-	-	0.23	-
[59]	10	28-97	0-5.6	51.16	22.74	11.76	1.57	2.54	6.19	1.84	1.59	-	-
[63]	14	100	2.9-9.7	34.52	8.35	11.23	13.02	8.05	-	-	-	7.31	-
[64]	4	33	1.1-2	51.16	14.01	11.17	6.34	4.88	2.16	2.76	3.81	-	-
[70]	20	10-40	0.1-3	40.80	10.60	15.00	16.00	10.80	1.11	-	1.72	-	-
[73]	5	27-43	14-19.1	69.25	4.78	14.37	7.77	0.83	1.39	-	1.34	0.31	-

Notes:
n: number of specimens; IOT: Iron Ore Tailings; LOI: Loss on Ignition; NS: The study reported the oxides content of two different IOT but did not specify to which water absorption values they corresponded.
Chemical composition reported in the studies and obtained by X-ray Fluorescence (XRF).

Table S9. Correlation and linear regression analysis between IOT chemical profile and water absorption.

Chemical Composition	n	Pearson r	Pearson p	Spearman ρ	Spearman p	Slope	Intercept	R^2	Interpretation
SiO_2	157	0.07	0.412	0.03	0.736	0.039	7.129	0.004	Positive association, not statistically significant
Al_2O_3	157	-0.13	0.096	-0.16	0.042	-0.157	10.445	0.018	Negative association, statistically significant
Fe_2O_3	157	0.13	0.094	0.21	0.008	0.061	7.037	0.018	Positive association, statistically significant
CaO	136	-0.28	< 0.001	-0.40	< 0.001	-0.365	11.468	0.079	Negative association, statistically significant
MgO	107	-0.36	< 0.001	-0.57	< 0.001	-0.764	12.420	0.129	Negative association, statistically significant
K_2O	124	-0.40	< 0.001	-0.52	< 0.001	-2.113	12.225	0.158	Negative association, statistically significant

Chemical Composition	n	Pearson r	Pearson p	Spearman ρ	Spearman p	Slope	Intercept	R^2	Interpretation
TiO_2	57	0.46	< 0.001	0.23	0.083	1.400	5.627	0.211	Positive association, not statistically significant
Na_2O	75	-0.36	0.002	-0.41	< 0.001	-3.608	13.150	0.127	Negative association, statistically significant
SO_3	51	-0.20	0.151	-0.13	0.347	-0.397	8.766	0.042	Negative association, not statistically significant
LOI	67	-0.15	0.214	-0.15	0.228	-0.327	13.792	0.024	Negative association, not statistically significant
SiO_2/Fe_2O_3	157	0.56	< 0.001	0.55	< 0.001	6.636	-1.155	0.317	Positive association, statistically significant
SiO_2/Al_2O_3	157	0.23	0.003	0.58	< 0.001	0.551	7.930	0.054	Positive association, statistically significant

Note:
n = number of observations available per chemical composition variable (varies due to incomplete reporting across studies); r = Pearson correlation coefficient; ϱ = Spearman's rank correlation coefficient; p = two-tailed probability value; R^2 = coefficient of determination (r^2).
* Statistical significance (α = 0.05) was assessed using Spearman's ϱ, given the scattered, non-normally distributed nature of the water absorption data.

References

1. Almada, B.S.; Da Silva Silveira Melo, H.; Duarte, M.S.; Aguilar, M.T.P.; Garcia, D.C.S.; Silva, G.J.B.; Dos Santos, W.J. Study of Mechanical, Durability and Microstructural Properties of Cementitious Composite with Addition of Different Iron Ore Tailings from Brazil. *Journal of Materials Research and Technology* **2022**, *18*, 1947–1962, doi:10.1016/j.jmrt.2022.03.070.
2. Babatunde, A.Q.; Oshioname, E.A.; Paul, Y.; Junwolo, O.K. Effect of Elapsed Time after Mixing on the Strength Properties of Lime–Iron Ore Tailings Treated Black Cotton Soil as a Road Construction Material. *Infrastructures* **2020**, *5*, 89, doi:10.3390/infrastructures5110089.
3. Bastos, L.A.D.C.; Silva, G.C.; Mendes, J.C.; Peixoto, R.A.F. Using Iron Ore Tailings from Tailing Dams as Road Material. *J. Mater. Civ. Eng.* **2016**, *28*, 04016102, doi:10.1061/(ASCE)MT.1943-5533.0001613.
4. Borges, P.H.R.; Ramos, F.C.R.; Caetano, T.R.; Panzerra, T.H.; Santos, H. Reuse of Iron Ore Tailings in the Production of Geopolymer Mortars. *REM, Int. Eng. J.* **2019**, *72*, 581–587, doi:10.1590/0370-44672017720169.
5. Carrasco, E.V.M.; Mantilla, J.N.R. Performance of Iron Ore Tailings/Cement Composite at High Temperatures: Inconbustibility. *RGSA* **2024**, *18*, e07665, doi:10.24857/rgsa.v18n3-158.
6. Carrasco, E.V.M.; Magalhaes, M.D.C.; Santos, W.J.D.; Alves, R.C.; Mantilla, J.N.R. Characterization of Mortars with Iron Ore Tailings Using Destructive and Nondestructive Tests. *Constr Build Mater* **2017**, *131*, 31–38, doi:10.1016/j.conbuildmat.2016.11.065.
7. Carvalho, J.V.D.A.; Wagner, A.C.; Scheuermann Filho, H.C.; Chaves, H.M.; Silva, J.P.S.; Delgado, B.G.; Consoli, N.C. Evaluation of Strength Parameters for Application in Cemented Iron Ore Tailings Stacks. *Indian Geotech J* **2023**, *53*, 775–788, doi:10.1007/s40098-023-00712-9.
8. Cechin, L.; Mymrine, V.; Avanci, M.A.; Povaluk, A.E. Ceramics Composites from Iron Ore Tailings and Blast Furnace Slag. *Ceramics International* **2022**, *48*, 10506–10515, doi:10.1016/j.ceramint.2021.12.260.
9. Bangalore Chinnappa, G.; Karra, R.C. Experimental and Statistical Evaluations of Strength Properties of Concrete with Iron Ore Tailings as Fine Aggregate. *J. Hazard. Toxic Radioact. Waste* **2020**, *24*, 04019038, doi:10.1061/(ASCE)HZ.2153-5515.0000480.
10. Consoli, N.C.; Lotero, A.; Scheuermann Filho, H.C.; Khajeh, A.; Daassi-Gli, C.P.A.; Vogt, J.C.; Silva, J.P.D.S. Effect of Cement Type on Compacted Iron Ore Tailings-Binder Response Blends: Comparative Study. *J. Mater. Civ. Eng.* **2024**, *36*, 04024230, doi:10.1061/JMCEE7.MTENG-17486.
11. Consoli, N.C.; Pedroso De Oliveira, J.; Lotero, A.; Scheuermann Filho, H.C.; Nuñéz, V. One-Part Alkali-Activated GGBFS as a Cement for Enhancing Compacted Filtered Iron Ore Tailings Disposal by Stacking. *Transportation Geotechnics* **2024**, *48*, 101306, doi:10.1016/j.trgeo.2024.101306.
12. El Khazanti, F.; Rachid, A.; El Ouahabi, M.; Nasri, H.; Azerkane, D.; Et-Tayea, Y.; Gharibi, E.K. Valorization of Iron Ore Tailings from Nador, Morocco, as a Sustainable Additive in the Manufacture of Red Clay Fired Bricks. *J. Ecol. Eng.* **2024**, *25*, 137–150, doi:10.12911/22998993/187956.
13. Etim, R.K.; Eberemu, A.O.; Osinubi, K.J. Stabilization of Black Cotton Soil with Lime and Iron Ore Tailings Admixture. *Transp. Geotech.* **2017**, *10*, 85–95, doi:10.1016/j.trgeo.2017.01.002.
14. Eugenio, T.M.C.; Henriques, A.B.; Mendes, R.F. Use of Iron Ore Tailings as Partial Replacement for Cement on Cementitious Composites Production with Vegetable Fibers. *Construction and Building Materials* **2024**, *411*, 134667, doi:10.1016/j.conbuildmat.2023.134667.
15. Farenzena, H.P.; Bruschi, G.J.; Medina, G.S.; de Sousa Silva, J.P.; Lotero, A.; Consoli, N.C. Iron Ore Tailings Stabilization with Alternative Alkali-Activated Cement for Dry Stacking: Mechanical and Microstructural Insights. *Can. Geotech. J.* **2024**, *61*, 649–667, doi:10.1139/cgj-2023-0125.
16. Fontes, W.C.; Franco De Carvalho, J.M.; Defaveri, K.; Brigolini, G.J.; Segadães, A.M.; Peixoto, R.A.F. Hydraulic Tiles Produced with Fine Aggregates and Pigments Reclaimed from Iron Ore Tailings. *J. Sustain. Metall.* **2021**, *7*, 151–165, doi:10.1007/s40831-020-00329-9.
17. Fontes, W.C.; Mendes, J.C.; Silva, S.N.D.; Peixoto, R.A.F. Mortars for Laying and Coating Produced with Iron Ore Tailings from Tailing Dams. *Construction and Building Materials* **2016**, *112*, 988–995, doi:10.1016/j.conbuildmat.2016.03.027.
18. De Freitas, S.M.A.C.; Sousa, L.N.; Diniz, P.; Martins, M.E.; Assis, P.S. Steel Slag and Iron Ore Tailings to Produce Solid Brick. *Clean Techn Environ Policy* **2018**, *20*, 1087–1095, doi:10.1007/s10098-018-1513-7.
19. Galvão, J.L.B.; Andrade, H.D.; Brigolini, G.J.; Peixoto, R.A.F.; Mendes, J.C. Reuse of Iron Ore Tailings from Tailings Dams as Pigment for Sustainable Paints. *Journal of Cleaner Production* **2018**, *200*, 412–422, doi:10.1016/j.jclepro.2018.07.313.
20. Gong, L.; Gong, X.; Liang, Y.; Jia, Z.; Li, Y. Experimental Study and Microscopic Analysis on Frost Resistance of Iron Ore Tailings Recycled Aggregate Concrete. *Advances in Materials Science and Engineering* **2022**, *2022*, 1–16, doi:10.1155/2022/8932229.

21. Gu, X.; Li, X.; Zhang, W.; Gao, Y.; Kong, Y.; Liu, J.; Zhang, X. Effects of HPMC on Workability and Mechanical Properties of Concrete Using Iron Tailings as Aggregates. *Materials* **2021**, *14*, 6451, doi:10.3390/ma14216451.
22. Gu, X.; Li, Z.; Zhang, Y.; Zhang, W.; Li, X.; Liu, B. Sustainable Assessment and Synergism of Ceramic Powder and Steel Slag in Iron Ore Tailings-Based Concrete. *Environ Sci Pollut Res* **2024**, *31*, 18856–18870, doi:10.1007/s11356-024-32396-w.
23. Guedes, J.P.C.; Wagner, A.C.; Carvalho, J.V.D.A.; Daassi-Gli, C.A.P.; Scheuermann Filho, H.C.; Consoli, N.C. Fibers Enhancing New Mine Waste-Based Alkaline-Activated Cement for Dry Stacking Purposes. *J. Mater. Civ. Eng.* **2024**, *36*, 04024151, doi:10.1061/JMCEE7.MTENG-17386.
24. Han, G.; Zhang, J.; Sun, H.; Shen, D.; Wu, Z.; An, X.; Meye, S.M.; Huang, Y. Application of Iron Ore Tailings and Phosphogypsum to Create Artificial Rockfills Used in Rock-Filled Concrete. *Buildings* **2022**, *12*, 555, doi:10.3390/buildings12050555.
25. Huang, S.; Cai, C.; Xu, J.; Zhu, S.; Pi, Z.; Li, H. Experimental Investigation on the Performance of Cement Mortar by Partial Replacement of the Standard Sands with Sulfide-Contained Iron Tailings. *J. Mater. Civ. Eng.* **2024**, *36*, 04024141, doi:10.1061/JMCEE7.MTENG-17014.
26. Jahanshahi, F.S.; Ghanizadeh, A.R. Compressive Strength, Durability, and Resilient Modulus of Cement-Treated Magnetite and Hematite Iron Ore Tailings as Pavement Material. *Construction and Building Materials* **2024**, *447*, 138076, doi:10.1016/j.conbuildmat.2024.138076.
27. Jing, C.; Shi, W.; Wang, N.; Xue, Z.; Zhao, Q.; Meng, X. Laboratory Investigation of Solid Wastes Combined with Tunnel Slag in Cement Stabilized Base of Asphalt Pavement. *Construction and Building Materials* **2023**, *392*, 131807, doi:10.1016/j.conbuildmat.2023.131807.
28. Kakodkar, S.A.; Sawaiker, U.G. High-Performance, Eco-Friendly Blocks from Iron Ore Tailings: A Solution for Sustainable Construction. *Nat. Env. Poll. Tech* **2025**, *24*, B4220, doi:10.46488/NEPT.2025.v24i01.B4220.
29. Kang, X.; Li, Y.; Li, W.; Zhou, Y.; Cui, J.; Cai, B.; Zi, Y.; Fang, J.; Chen, Y.; Li, S.; et al. Synthesis and Characterization of Sustainable Eco-Friendly Alkali-Activated High-Content Iron Ore Tailing Bricks. *Buildings* **2023**, *13*, 2743, doi:10.3390/buildings13112743.
30. Khamseh, B.; Shourijeh, P.T.; Binesh, S.M. Strength and Deformation Characteristics of Cement-Stabilized Fe-Rich Fine Iron Ore Tailings. *Construction and Building Materials* **2025**, *463*, 140101, doi:10.1016/j.conbuildmat.2025.140101.
31. Kumar, G.S.; Gayathri Nivedha, R.; Venkatesha, G.; Ismail, M.; Sridhar, H.N.; Ramaraju, H.K. Laboratory Investigation of Workability and Mechanical Properties of Concrete Utilizing Fly Ash and Iron Ore Tailing Waste. *Innov. Infrastruct. Solut.* **2022**, *7*, 302, doi:10.1007/s41062-022-00906-9.
32. Li, H.-N.; Liu, P.-F.; Li, C.; Li, G.; Zhang, H. Experimental Research on Dynamic Mechanical Properties of Metal Tailings Porous Concrete. *Construction and Building Materials* **2019**, *213*, 20–31, doi:10.1016/j.conbuildmat.2019.04.049.
33. Li, H.; Liu, F.; Zhen, H.; Xiong, Z.; Song, Y.; Wang, J.; Li, L. Evaluating Strength, Hydration Characteristics, Microstructure Evolution, and Sustainability of Seawater–Sea Sand Cement-Based Materials Containing Iron Ore Tailings. *Construction and Building Materials* **2024**, *457*, 139163, doi:10.1016/j.conbuildmat.2024.139163.
34. Li, L.; Jiang, T.; Zhou, M.; Chen, B.; Chen, C. The Influence of Temperature and SiC Content on the Recycling of Iron Ore Tailings for the Preparation of Value-Added Foam Ceramics. *J Mater Cycles Waste Manag* **2021**, *23*, 330–340, doi:10.1007/s10163-020-01135-x.
35. Liu, K.; Wang, S.; Quan, X.; Jing, W.; Xu, J.; Zhao, N.; Liu, B.; Ying, H. Industrial Byproduct Iron Ore Tailings as Ecofriendly Materials in the Utilization of Cementitious Composites. *Construction and Building Materials* **2023**, *372*, 130813, doi:10.1016/j.conbuildmat.2023.130813.
36. Luan, C.; Liu, J.; Zhao, S.; Li, Y.; Yang, Y. Enhancing the Properties of UHPC with Recycled Concrete Powder and Iron Ore Tailings Sand, and Evaluating the Environmental Impact. *Construction and Building Materials* **2024**, *452*, 138769, doi:10.1016/j.conbuildmat.2024.138769.
37. Mafessoli, M.; Marques, S.F.V.; Scheuermann Filho, H.C.; Consoli, N.C. Response of Artificially Cemented Iron Ore Tailings for Dry Stacking Disposal over a Wide Range of Stresses. *Indian Geotech J* **2023**, *53*, 904–915, doi:10.1007/s40098-023-00711-w.
38. Mazzinghy, D.B.; Figueiredo, R.A.M.; Parbhakar-Fox, A.; Yahyaei, M.; Vaughan, J.; Powell, M.S. Trialling One-Part Geopolymer Production Including Iron Ore Tailings as Fillers. *International Journal of Mining, Reclamation and Environment* **2022**, *36*, 356–367, doi:10.1080/17480930.2022.2047271.
39. Mendes, B.C.; Pedroti, L.G.; Fontes, M.P.F.; Ribeiro, J.C.L.; Vieira, C.M.F.; Pacheco, A.A.; Azevedo, A.R.G.D. Technical and Environmental Assessment of the Incorporation of Iron Ore Tailings in Construction Clay Bricks. *Construction and Building Materials* **2019**, *227*, 116669, doi:10.1016/j.conbuildmat.2019.08.050.

40. Mymrin, V.; Pedroso, C.L.; Pedroso, D.E.; Rolim, P.H.B.; Catai, R.E.; Carvalho, K.Q. Eco-Friendly Cementless Concrete from Hazardous Iron Ore Tailing and Waste of Cellulose-Pulp, Concrete Demolition, and Lime Production. *Int J Adv Manuf Technol* **2022**, *122*, 933–945, doi:10.1007/s00170-022-09811-y.
41. Mymrin, V.; Pedroso, D.E.; Pedroso, C.L.; Avanci, M.A.; Rolim, P.H.B.; Carvalho, K.Q.; Catai, R.E. Physical-Chemical Processes of Sustainable Construction Materials Structure Formation with Iron Ore Processing Tailings and Aluminum Anodizing Sludge. *Construction and Building Materials* **2021**, *298*, 123698, doi:10.1016/j.conbuildmat.2021.123698.
42. Okoro, I.J.; Amadi, A.A.; Alhassan, M. Hydraulic Conductivity Characteristics of Iron-Ore Tailings-Bentonite Mixtures Exposed to Landfill Leachate. *J. Hazard. Toxic Radioact. Waste* **2023**, *27*, doi:10.1061/JHTRBP.HZENG-1187.
43. Osinubi, K.J.; Yohanna, P.; Eberemu, A.O. Cement Modification of Tropical Black Clay Using Iron Ore Tailings as Admixture. *Transp. Geotech.* **2015**, *5*, 35–49, doi:10.1016/j.trgeo.2015.10.001.
44. Rao, P.S.; Chandar, K.R. Development of Energy Efficient Organic Bricks in Construction Using IOT and Perlite. *International Journal of Sustainable Engineering* **2021**, *14*, 865–873, doi:10.1080/19397038.2020.1838662.
45. Reddy, B.V.V.; Hemanth Kumar, H.; Ullas, S.N.; Gourav, K. Non-Organic Solid Wastes - Potential Resource for Construction Materials. *Current Science* **2016**, *111*, 1968, doi:10.18520/cs/v111/i12/1968-1976.
46. Sá, T.S.W.; Oda, S.; Karla Castelo Branco Louback Machado Balthar, V.; Dias Toledo Filho, R. Use of Iron Ore Tailings and Sediments on Pavement Structure. *Construction and Building Materials* **2022**, *342*, 128072, doi:10.1016/j.conbuildmat.2022.128072.
47. Silva, H.E.D.; Antoniosi Filho, N.R. Remediation of Iron Ore Tailings From Brumadinho (Brazil), Polymeric Waste From Polyester Fabrics and From PET Bottle Powder Based on the Production of Ecological Bricks. *Remediation Journal* **2025**, *35*, e70012, doi:10.1002/rem.70012.
48. Silva, C.B.D.; Paiva, P.R.P.D. Artificial Stone Production Using Iron Ore Tailing (IOT). *Cerâmica* **2020**, *66*, 164–171, doi:10.1590/0366-69132020663782854.
49. Singh, R.K.; Debjit Pal; Shailendra Kr Signh; Nimisha Tripathi; Raj S Singh Utilization of Iron Ore Tailings for Brick Manufacture from Donimalai Mines of Karnataka, India. *JSIR* **2021**, *80*, doi:10.56042/jsir.v80i03.41609.
50. Sridhar, H.N.; Kumar, G.S.; Chandan, K.; Ramaraju, H.K. Experimental Investigation of the Engineering Properties of Stabilized Black Cotton Soil Using Industrial Waste Materials. *Transp. Infrastruct. Geotech.* **2025**, *12*, doi:10.1007/s40515-024-00477-5.
51. Subramanian, S.; Davis, R.; Thomas, B.S. Microstructure and Residual Strength Properties of Engineered Geopolymer Composites (EGC) Subjected to High Temperatures. *Journal of Building Engineering* **2024**, *96*, 110637, doi:10.1016/j.jobe.2024.110637.
52. Subramanian, S.; P, R.D.; Thomas, B.S. Sustainable Engineered Geopolymer Composites: A Study on the Potential of Fly Ash, BOF Slag, and Iron Ore Tailings. *Environmental Engineering Research* **2024**, *29*, 230540–0, doi:10.4491/eer.2023.540.
53. Tang, Z.; Meng, X.; Han, Y.; Chen, M.; Gao, P.; Zhang, Y. Hydration and Properties of Hydrogen-Based Mineral Phase Transformation Iron Ore Tailings as Supplementary Cementitious Material. *Journal of Environmental Management* **2024**, *356*, 120594, doi:10.1016/j.jenvman.2024.120594.
54. Thejas, H.K.; Hossiney, N. Alkali-Activated Bricks Made with Mining Waste Iron Ore Tailings. *Case Studies in Construction Materials* **2022**, *16*, e00973, doi:10.1016/j.cscm.2022.e00973.
55. Thejas, H.K.; Hossiney, N. Compressed Unfired Blocks Made with Iron Ore Tailings and Slag. *Cogent Engineering* **2022**, *9*, 2032975, doi:10.1080/23311916.2022.2032975.
56. Tian, X.; Wang, C.; Asrah, H.; Han, L.C.; Zheng, Y. Investigating the Grinding Characteristics of Vanadium-Titanium Iron Ore Tailings for Sustainable Utilization in Cementitious Material Preparation. *Alexandria Engineering Journal* **2024**, *101*, 78–89, doi:10.1016/j.aej.2024.05.057.
57. Vescovi, P.H.T.; Bezerra, A.C.D.S.; Poggiali, F.S.J. Iron Ore Tailing (IOT) Chemical Composition and Content Impact on the Physical, Mechanical, and Durability Properties of Blended Cement. *Journal of Building Engineering* **2023**, *80*, 108119, doi:10.1016/j.jobe.2023.108119.
58. Wang, S.; Gao, Y.; Liu, K. Mechanical Properties and Sulfate Freeze–Thaw Resistance of Calcium Carbonate Whisker-Reinforced Iron Ore Tailings Concrete. *Buildings* **2024**, *14*, 1004, doi:10.3390/buildings14041004.
59. Wang, L.; Tian, H.; Lei, W.; Dai, N.; Wang, H. Development of High-Strength Ceramsite via Sintering of Iron Ore Tailings: Process Optimization and Properties. *Construction and Building Materials* **2024**, *457*, 139440, doi:10.1016/j.conbuildmat.2024.139440.
60. Wang, T.; Ning, X.; Yang, L.; Rao, F.; Jiang, K. The Synthesis of an Iron Tailings-Based Geopolymer with Synergistic Electromagnetic Wave Consumption Property. *Environmental Research* **2025**, *268*, 120744, doi:10.1016/j.envres.2024.120744.

61. Wang, T.; Rao, F.; Yang, L.; Jiang, K.; Lin, N.; Mo, L. Influence of Wastes and Synthesis Conditions on the Compressive Strength, Setting Time and Gels of Waste-Based Geopolymers. *Gels* **2024**, *10*, 700, doi:10.3390/gels10110700.
62. Wei, Z.; Jia, Y.; Wang, S.; Li, Z.; Li, Y.; Wang, X.; Gao, Y. Utilization of Iron Ore Tailing as an Alternative Mineral Filler in Asphalt Mastic: High-Temperature Performance and Environmental Aspects. *Journal of Cleaner Production* **2022**, *335*, 130318, doi:10.1016/j.jclepro.2021.130318.
63. Weishi, L.; Guoyuan, L.; Ya, X.; Qifei, H. The Properties and Formation Mechanisms of Eco-Friendly Brick Building Materials Fabricated from Low-Silicon Iron Ore Tailings. *Journal of Cleaner Production* **2018**, *204*, 685–692, doi:10.1016/j.jclepro.2018.08.309.
64. Yan, Y.; Du, X.; Kuang, Y. Study on the Influence of Nickel-Iron Alloy Powder on the Mechanical Strength and Electromagnetic Shielding Effectiveness of Iron Ore Tailings/Cement Composite. *Case Studies in Construction Materials* **2024**, *21*, e03740, doi:10.1016/j.cscm.2024.e03740.
65. Yang, G.; Liu, F.; Xie, Q.; Yang, M.; Li, Y.; Kumar, E.R.; Sun, J. Exploration of Iron Ore Tailings with High Volume in Sustainable Cement and Ecofriendly Cementitious Material. *Developments in the Built Environment* **2024**, *19*, 100482, doi:10.1016/j.dibe.2024.100482.
66. Zhang, Y.; Li, Z.; Gu, X.; Nehdi, M.L.; Marani, A.; Zhang, L. Utilization of Iron Ore Tailings with High Volume in Green Concrete. *Journal of Building Engineering* **2023**, *72*, 106585, doi:10.1016/j.jobe.2023.106585.
67. Zhang, Y.; Liu, B.; Gu, X.; Nehdi, M.L.; Zhang, L.V. Mechanochemical Activation of Iron Ore Tailing-Based Ternary Supplementary Cementitious Materials. *Construction and Building Materials* **2022**, *346*, 128420, doi:10.1016/j.conbuildmat.2022.128420.
68. Zhang, Y.; Yang, D.; Gu, X.; Chen, H.; Li, Z. Application of Iron Tailings-Based Composite Supplementary Cementitious Materials (SCMs) in Green Concrete. *Materials* **2022**, *15*, 3866, doi:10.3390/ma15113866.
69. Zhang, Y.; Zhang, L.; Wang, Q.; Han, D.; Li, Z. Iron Ore Tailings, Phosphate Slags, and Lithium Slags as Ternary Supplementary Cementitious Materials for Concrete: Study on Compression Strength and Microstructure. *Materials Today Communications* **2023**, *36*, 106644, doi:10.1016/j.mtcomm.2023.106644.
70. Zhang, C.; Ren, Z.; Wang, L.; Zhang, N.; Li, X.; Han, X.; Liang, J. The Synergistic Sintering of Ultra-Lightweight Ceramsite from Molybdenum Ore Tailings, Iron Ore Tailings and Waste Glass Powders: Properties and Formation Mechanism. *Construction and Building Materials* **2024**, *452*, 138852, doi:10.1016/j.conbuildmat.2024.138852.
71. Zhang, H.; Qi, Y.; Jing, J.; Wang, C.; Zhou, Y.; Zhang, K.; Zheng, Y.; Zhai, Y.; Liu, F. Properties and Environmental Impact of Building Foundation Pit Backfilling Materials Containing Iron and Steel Solid Waste. *Front. Earth Sci.* **2023**, *11*, 1181974, doi:10.3389/feart.2023.1181974.
72. Zhao, J.; Su, Y.; Shi, Y.; Wang, Q.; Ni, K. Study on Mechanical Properties of Macro-synthetic Fiber-reinforced Iron Ore Tailings Concrete. *Structural Concrete* **2022**, *23*, 423–440, doi:10.1002/suco.202100383.
73. Zhong, M.; Meng, J.; Ning, B.; Na, F.; Cui, T.; Shi, X.; Cui, T. Preparation and Alkali Excitation Mechanism of Coal Gangue-Iron Ore Tailings Non-Sintering Ceramsite. *Construction and Building Materials* **2024**, *426*, 136209, doi:10.1016/j.conbuildmat.2024.136209.
74. Zhou, H.; Jiang, Y.; Wang, J.; Zhang, H.; Qian, B.; Ma, B.; Hu, Y. The Composition and Performance of Iron Ore Tailings in Steel Slag-Based Autoclaved Aerated Concrete. *Buildings* **2023**, *13*, 2942, doi:10.3390/buildings13122942.
